# Delineating functional and molecular impact of ex vivo sample handling in precision medicine

**DOI:** 10.1038/s41698-024-00528-7

**Published:** 2024-02-19

**Authors:** Nona Struyf, Albin Österroos, Mattias Vesterlund, Cornelia Arnroth, Tojo James, Stephanie Sunandar, Georgios Mermelekas, Anna Bohlin, Kerstin Hamberg Levedahl, Sofia Bengtzén, Rozbeh Jafari, Lukas M. Orre, Janne Lehtiö, Sören Lehmann, Päivi Östling, Olli Kallioniemi, Brinton Seashore-Ludlow, Tom Erkers

**Affiliations:** 1 Department of Oncology-Pathology, Karolinska Institutet, Science for Life Laboratory, Stockholm, Sweden; 2 Department of Medical Sciences, Uppsala University Hospital, Uppsala, Sweden; 3 Department of Medicine, Center for Hematology and Regenerative Medicine, Karolinska Institute, Stockholm, Sweden; 4 Department of Public Health and Caring Sciences, Uppsala University, Uppsala, Sweden; 5 Institute for Molecular Medicine Finland, University of Helsinki, Helsinki, Finland

**Keywords:** Acute myeloid leukaemia, High-throughput screening, Proteomic analysis

## Abstract

Consistent handling of samples is crucial for achieving reproducible molecular and functional testing results in translational research. Here, we used 229 acute myeloid leukemia (AML) patient samples to assess the impact of sample handling on high-throughput functional drug testing, mass spectrometry-based proteomics, and flow cytometry. Our data revealed novel and previously described changes in cell phenotype and drug response dependent on sample biobanking. Specifically, myeloid cells with a CD117 (c-KIT) positive phenotype decreased after biobanking, potentially distorting cell population representations and affecting drugs targeting these cells. Additionally, highly granular AML cell numbers decreased after freezing. Secondly, protein expression levels, as well as sensitivity to drugs targeting cell proliferation, metabolism, tyrosine kinases (e.g., JAK, KIT, FLT3), and BH3 mimetics were notably affected by biobanking. Moreover, drug response profiles of paired fresh and frozen samples showed that freezing samples can lead to systematic errors in drug sensitivity scores. While a high correlation between fresh and frozen for the entire drug library was observed, freezing cells had a considerable impact at an individual level, which could influence outcomes in translational studies. Our study highlights conditions where standardization is needed to improve reproducibility, and where validation of data generated from biobanked cohorts may be particularly important.

## Introduction

Functional testing and molecular monitoring ex vivo are widely implemented to gain insight into molecular mechanisms in cancer and represent future opportunities for precision oncology^1,2^. In hematology, mononuclear cells (MNCs) from bone marrow (BM) or peripheral blood (PB) are accessible and provide large quantities of cancer cells for data generation^3^. Biobanking of patient MNCs is a crucial part of translational research, as it is usually standard practice at larger cancer centers^4^. However, there is currently no standard procedure for handling and storing samples for functional and molecular profiling^5,6^. When comparing sample handling between studies using functional testing on blood cancer cells, the main variation in protocols arises from the use of fresh or biobanked (frozen) samples (Supplementary Fig. 1a; Supplementary Table 1). There is a clear need for method standardization to increase consistency between laboratories and augment the potential for functional precision medicine and biomarker discovery^7^.

**Fig. 1:**
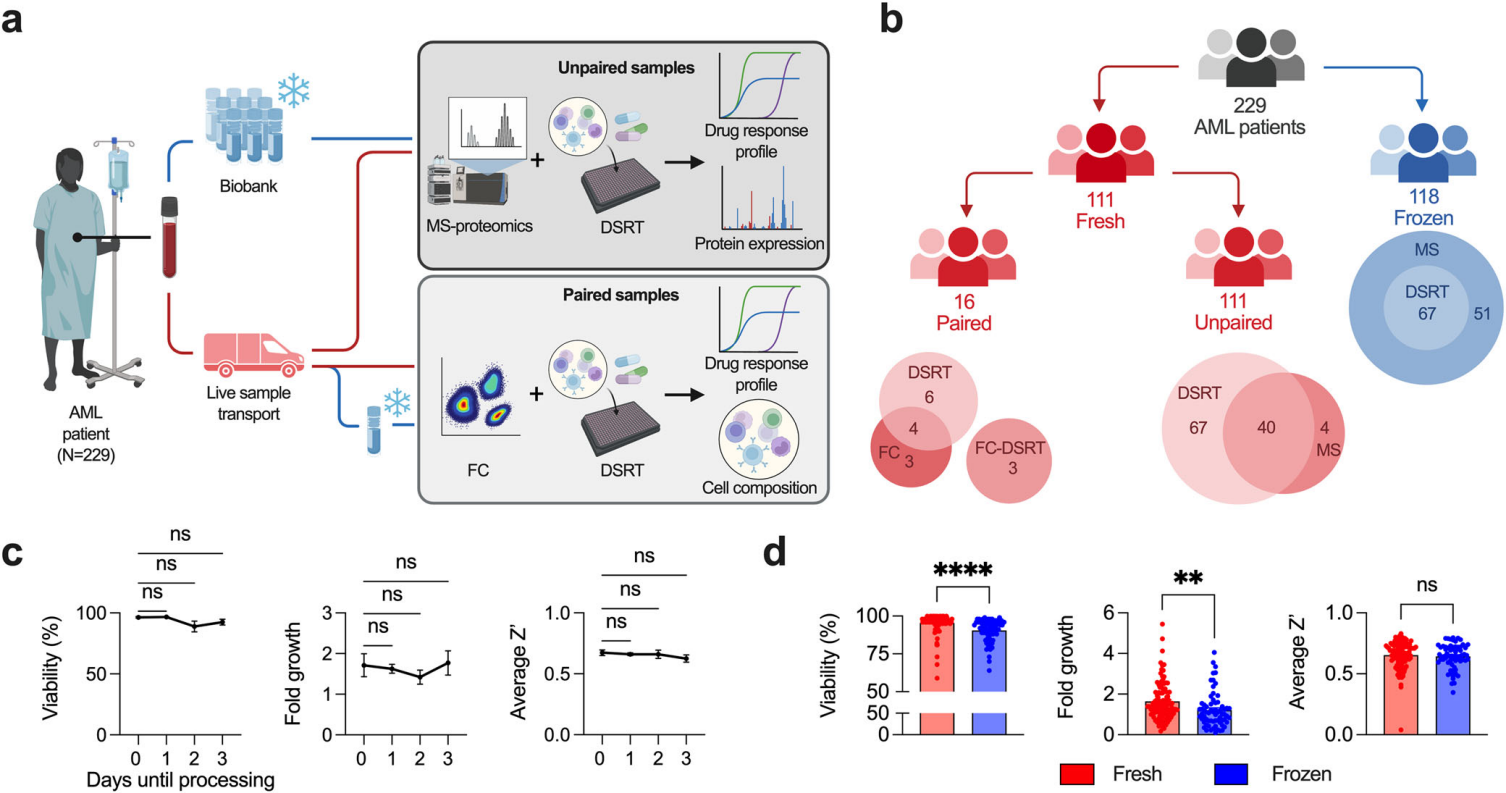
Cryopreservation reduces viability but does not affect assay quality. **a**, **b** Visual overview of the cohorts and methods, created with BioRender. **c** A comparison of several parameters such as cell viability after isolation or thawing, fold growth (cell proliferation) during the assay, and assay quality (Z’) for samples processed at different timepoints from sample acquisition (day 0: *n* = 18, day 1: *n* = 69, day 2: *n* = 11, day 3: *n* = 13), **d** and for fresh (*n* = 111) and frozen (*n* = 67) samples. Mean with SEM are shown using Kruskal–Wallis and Welch’s *t* test, respectively, *P ≤ 0.05, ****P ≤ 0.0001, ns = not significant. DSRT indicates drug sensitivity and resistance testing; FC, flow cytometry; MS, mass spectrometry-based proteomics; FC-DSRT, flow cytometry-based drug sensitivity and resistance testing.

In the present study, we asked if functional testing and phenotypic profiling outcomes are dependent on sample handling, such as biobanking and time from sample draw until data generation. To do this, we leveraged ex vivo drug sensitivity and resistance testing (DSRT, *n* = 528 drugs) and mass spectrometry (MS)-based proteomics (*n* = 10,648 proteins) in an AML patient cohort consisting of 111 fresh and 118 biobanked samples (Fig. 1a, b). In addition, we performed a systematic study using 16 paired samples for DSRT (*n* = 7), flow cytometry (FC)-based phenotyping (*n* = 10) and FC-based DSRT (*n* = 3).

## Cryopreservation induces changes in molecular phenotypes and functional drug responses

First, we investigated if sample transport time up to three days from draw until processing affected the DSRT, but no differences could be observed (Fig. 1c). Both cell viability and fold growth were lower for the biobanked cohort, while the Z’ score, an assay quality parameter^8^, showed no difference (Fig. 1d). For samples which had been biobanked for a longer time, cell yield was slightly reduced (Supplementary Fig. 1b). Both fresh and frozen cohorts had overall similar clinical, cytogenetic, and molecular characteristics (Supplementary Tables 2–4). After correcting for age and sex the main differences were a higher rate of AML patients with antecedent hematological disease in both frozen cohorts, and an increase in median age, as well as *NRAS* and *RAD21* mutational status in the fresh MS-proteomic cohort.

**Fig. 2:**
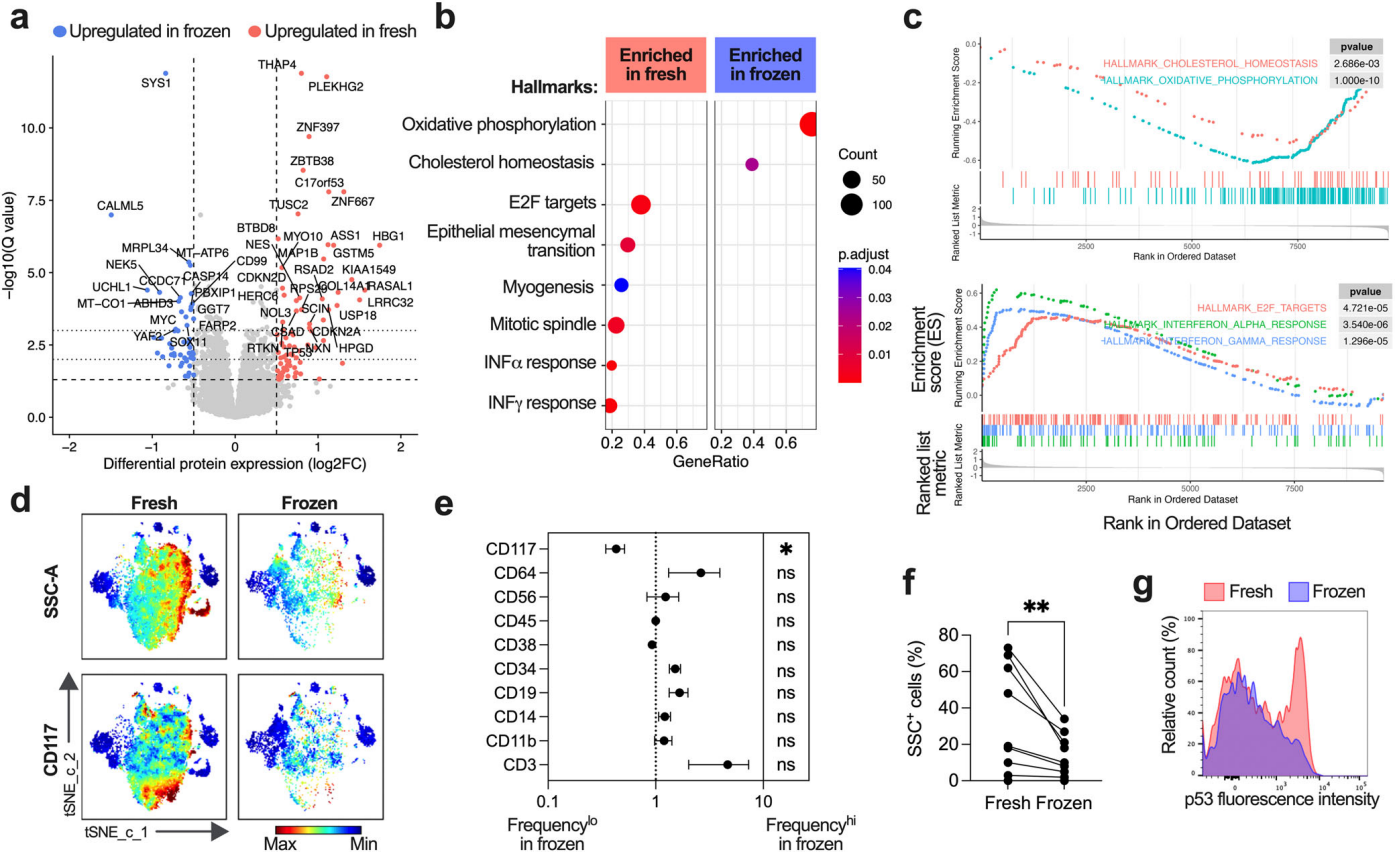
Cryopreservation upregulates stress related proteins while reducing p53 expression, as well as CD117^+^ and SSC^+^ cells. **a** A volcano plot showing all tested proteins with cutoff lines shown at 0.5 log2FC and *q* values of 0.05, 0.01 and 0.001, false discovery rate was set to 0.05. Significantly differentially expressed proteins (*Q* < 0.05) in fresh (*n* = 44) and frozen (*n* = 118) are shown in blue and red, respectively. **b** Gene sets which are significantly enriched in fresh or frozen samples according to GSEA, color and size correlate to *P*-value and number of genes in the Gene Ontology (GO) term, respectively. GeneRatio is the percentage of differentially expressed genes within the GO term. **c** Enrichment plots showing the most significantly enriched gene sets in frozen (top) and fresh (bottom) samples. **d** A representative t-SNE plot (*n* = 1) visualizing the difference in marker expression and number of CD117^+^ and SSC^+^ cells before and after freezing. **e** Fold change in number of positive cells (*n* = 10) for each tested marker shown as mean with SEM, statistical significance was determined with paired *t* test, *P ≤ 0.05, ns = not significant. **f** Number of SSC^+^ cells before and after freezing (*n* = 10), significance tested with Wilcoxon test, **P ≤ 0.01. **g** A representative histogram showing relative intracellular p53 intensity before and after freezing.

Protein expression data for fresh and biobanked samples showed a difference in 820 out of 9692 analyzed proteins (Fig. 2a), including tumor suppressor protein p53 (*TP53*) which was decreased in frozen samples (0.650 ± 0.405, 0.012). In contrast, c-Myc (*MYC*) protein levels were increased in frozen samples (0.594 ± 0.254, 0.0003, Supplementary Table 5). Frozen samples also showed a relative reduction in proteins involved in cell cycle and cytokine signaling, and an increase in proteins related to oxidative stress and metabolism (Fig. 2b-c). Flow cytometry was then used to determine cell composition in patient-paired AML BM-MNCs over time, and before and after freezing (Supplementary Fig. 1c). A decrease in CD117^+^ (c-KIT) cells was observed over time (Supplementary Fig. 1d, e), and after freezing (Fig. 2d, e; Supplementary Fig. 1f, g). Fresh samples with high granularity also showed a decrease in SSC^hi^ (side-scatter) cells (Fig. 2f) and SSC mean fluorescence intensity (MFI) after freezing (Supplementary Fig. 1h). On the contrary, CD3^+^ T cells and CD38^-^CD34^+^ leukemic stem cells (LSC) were increased after freezing (Supplementary Fig. 1i). Moreover, a consistent decrease in p53 expression after freezing was also observed in patient-paired samples (Fig. 2g).

**Fig. 3:**
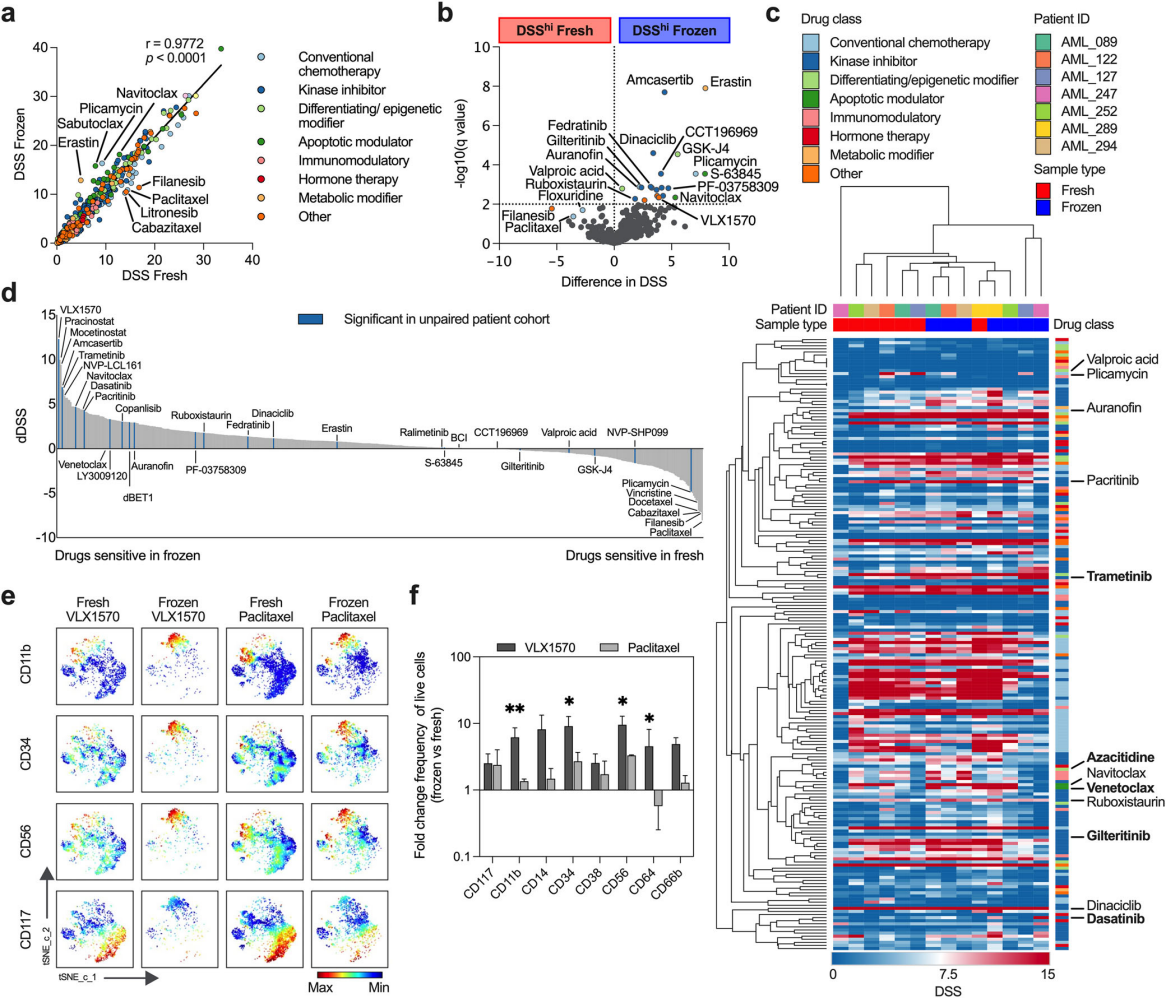
Drug responses and post-drug treatment phenotype affected by cryopreservation. **a** The drug response correlation between fresh (*n* = 107) and frozen (*n* = 67) samples, shown as average DSS for each drug with linear regression and Spearman’s rank correlation. **b** Multiple *t* tests showing 22 drugs with significant differences in fresh and frozen patients with a false discovery rate of 1%. **c** Comparison of patient-paired (*n* = 7) DSS values for 200 drugs before and after freezing, hierarchical clustering was done using one minus Spearman’s correlation based on the average value. Significant drugs from the unpaired cohort are highlighted, and drugs clinically used in AML are marked in bold. **d** Differential DSS (dDSS; mean DSS frozen–mean DSS fresh) is shown for the paired samples (*n* = 7), drugs which were significant in the unpaired cohort are marked in blue. **e** A t-SNE plot (*n* = 1) visualizing the difference in CD11b^+^, CD34^+^, CD56^+^ and CD117^+^ cells before and after drug treatment in paired fresh and frozen samples. **f** Fold change in number of positive cells for each marker after drug treatment in paired patients (*n* = 3) shown as mean with SEM and unpaired *t* tests. *P ≤ 0.05, **P ≤ 0.01, ns = not significant.

A good correlation in drug sensitivity score (DSS) was observed for DSRT performed after sample isolation on day one and two, whereas the correlation was poor after five days (Supplementary Fig. 2a). For fresh and frozen samples, a very high correlation was observed for the average response to each drug (Fig. 3a) and drug class (Supplementary Fig. 2b). However, 22 out of 528 drugs were more sensitive in the frozen cohort (Fig. 3b; Supplementary Fig. 2c; Supplementary Table 6).

Subsequently in the patient-paired cohort, drug response profiles clustered based on sample handling rather than by patient (Fig. 3c), suggesting that the systematic differences induced by biobanking are greater than the assumed biological similarity between two samples from the same patient. Out of the 22 affected drugs in the large cohort, 5 were also noticeably changed (dDSS > 4) in the patient-paired cohort. These include the BH3 mimetic navitoclax, the proteosome inhibitor VLX1570, the antineoplastic agent plicamycin, and the kinase inhibitors amcasertib and pacritinib. In addition, patient-paired samples showed changes in several other compounds. Increased sensitivity could be observed for tyrosine kinase inhibitors (trametinib, dasatinib), HDAC inhibitors (pracinostat, mocetinostat) and apoptotic modulators (NVP-LCL161, venetoclax) in frozen samples. Inversely, reduced sensitivity was observed for microtubule inhibitors (paclitaxel, cabazitaxel, vincristine, vinblastine) and kinesin inhibitors (filanesib, litronesib) (Fig. 3d, Supplementary Fig. 2d, e).

Moreover, patient-paired FC-based DSRT was used to investigate a selection of drugs based on the previous observations. Perturbation with the proteasome inhibitor VLX1570 and the microtubule inhibitor paclitaxel induced changes in marker expression in frozen samples compared to fresh samples (Fig. 3e, f; Supplementary Fig. 2f, g).

Lastly, we investigated if freezing would have an impact on translational outcomes. We used the molecular tumor board criteria from a study by Malani et al. to determine if the paired patients would receive a different treatment recommendation when using drug response profiles from fresh and frozen cells (Supplementary Fig. 3a)^1^. When looking at all 18 drugs which were translationally used in the study, one or more drug responses changed for each patient based on the sDSS cutoff criteria of 8.7 used by the authors (Supplementary Fig. 3b, c).

## Considerations for sample handling in molecular and functional testing studies

Sample handling is a key factor in studies involving human cells. Aspects like transport time, temperature, storage type, isolation methods, and culture medium could influence functional and molecular profiles and introduce systematic errors in generated data. In this study, we focus on evaluating the time until processing and the use of freshly acquired samples versus biobanked samples as these are the main variations between functional testing studies.

One major finding was that there is a consistent decrease in CD117^+^ cells after biobanking samples. CD117 or c-KIT is a tyrosine kinase (TK) receptor that is broadly expressed on both healthy hematopoietic cells and AML blasts. Overexpression of c-KIT in AML has been linked with an increase in proliferation, maturation inhibition and blocking of apoptosis. This observation is also reflected by the increased response of a TK inhibitor subset, including dasatinib, and proteasome deubiquitinase inhibitors (e.g., VLX-1570) after biobanking, all of which are known to also target c-KIT^9,10^. This finding could be of importance in studies where c-KIT is used as a selection marker for the leukemic cell population. One example of this approach is the study by Snijder et al. where image-based functional drug testing was performed, and drug scores were calculated for CD34^+^ or CD117^+^ cells^11^. Another observation was the reduction of SSC^hi^ cells after biobanking. Although the challenges of cryopreserving granulocytes are widely known^12^, here we also report the sensitivity to biobanking of mononuclear AML cells with high granularity. As shown earlier, this could give a false representation of the cell population frequencies in samples with a large granular AML cell population, as well as affect responses in drugs targeting these populations. For instance, the increased frequency of the T cell and LSC populations we observed are likely biased due to the decrease of the larger SSC^hi^ and CD117^+^ populations.

Our study shows that drug classes of high clinical importance for AML such as TK inhibitors, chemotherapy drugs, and BH3 mimetics are affected by cryopreservation. Interestingly, the biology that we determine to be affected by protein expression level and cell composition are in line with the drug target biology. For instance, the increase in sensitivity to BH3 mimetics and other apoptotic modulators is consistent with the increase in oxidative stress-related proteins, as well as with the decrease in p53 protein levels^13^. The increased sensitivity of the ferroptosis-inducing agent erastin in frozen samples was linked with decreased p53 protein levels, which is consistent with studies showing the relationship with p53 expression and ferroptosis inhibition^14,15^. Additionally, the proto-oncogene c-Myc (MYC), which serves an opposing role in cell regulation, was increased in frozen samples. Another example is the reduced response in conventional chemotherapy drugs and kinesin inhibitors after biobanking. This correlates with the observed fold growth decrease in the viability assay, as well as a relative reduction in proteins related to mitosis, since these drug classes target highly proliferating cells. These findings show that the shift in metabolism and protein expression after biobanking can considerably impact drug responses.

Previous studies have investigated the effects of cryopreservation on functional testing using smaller drug libraries in smaller patient cohorts. In a study by Degnin et al., 3 out of 19 tested TK inhibitors had significant differences in drug response after biobanking in 17 paired patients^16^. These results are consistent with some of the observations in our study. Another study by Meszaros et al. investigated differences between unpaired fresh and frozen samples for 139 compounds and found a strong correlation between both cohorts with a slight increase in sensitivity to drugs in biobanked samples^17^. However, this functional drug testing assay was performed on CD34^+^ cells, which in our observations are not significantly affected by freezing unless the CD34^+^CD38^−^ population was specifically selected. The authors also compared transcriptomic profiles and observed a downregulation of genes involved with cell proliferation and inflammation, which is concordant with our observations of the changes at the protein level. Aspects like culture conditions and culture medium impact on drug responses have been investigated in-depth previously^6^. Nevertheless, there is still a lack of knowledge on other aspects such as storage time and conditions. Our results indicate that the time from sample acquisition to cell isolation is important, and that drug response correlations, as well as CD117^+^ cells decrease with increased waiting time, implying that fresh samples should be utilized as soon as possible.

Our results show that caution should be used when using only frozen or a mix of fresh and frozen samples for functional testing, since it can introduce systematic errors. The use of frozen material is, however, more convenient with the existence of large biobanks. Currently, no good solution exists to normalize for the effects of freezing on functional drug testing studies. However, not all drugs are equally affected by freezing, implying that fresh and frozen datasets can be analyzed together depending on the drugs or proteins of interest. In other cases where the effects are noticeable, it would be best to use the cohorts separately.

To define the potential clinical impact of sample handling in functional precision medicine studies, we used the data from our paired cohort to compare all the drugs recommended by a molecular tumor board in a study by Malani et al. ^1^. For each patient in the paired cohort, at least one out of the 18 drugs recommended by a functional molecular tumor board changed to a degree that it would potentially influence treatment decision, were the cells to be biobanked before functional testing. This implies that while the correlation for the entire drug library is high, freezing cells and assay variability could have an impact on the individual and translational level.

## Limitations

Even though we show the importance of sample handling and the potential biases it may create in a dataset, there are limitations to this study, which highlight the need for further investigations at other centers. Despite optimizing and standardizing the assay, it is important to recognize the possibility of technical variations that may impact assay outcome. This could be mitigated by increasing the number of replicates and the dose point titration, at the expense of increasing the assay and sample size needed. Additional paired samples for drug testing and increased overlap of data types would increase the statistical power of the analyses. Moreover, in the MS-proteomics data where the cohorts were run separately, controls were added to normalize across batches. Thirdly, our flow cytometry panel was limited to commonly used myeloid and lymphoid markers, as well as key proteins selected based on the MS-proteomics analysis. Due to heterogenous nature of AML several other markers could potentially be affected by freezing. In addition, studies focusing on harmonization methods for functional testing data would increase the possibility for large scale analyses on merged datasets from multiple centers.

In conclusion, the present study describes how cryopreservation introduces substantial alterations to cell fitness and composition that propagates to molecular and functional testing outcomes. Thus, consideration of sample handling is of particular importance when assessing LSCs, CD117^+^ and highly granular leukemic target cells, as well as drugs affecting cell proliferation and metabolism, protein kinases (e.g., JAK, KIT and FLT3), and BH3 mimetics.

## Methods

### HS-5 conditioned medium

HS-5 conditioned medium (CM) was generated by incubating confluent HS-5 (ATCC, Manassas, VA) in complete RPMI medium consisting of RPMI 1640 (Sigma-Aldrich, St. Louis, MO), 10% FBS (Thermo Fisher), 2 mM L-glutamine (Sigma-Aldrich), 100 IU/mL Penicillin and 0.1 mg/mL Streptomycin (Pen-Strep, Sigma-Aldrich), for 72 h. The supernatant was filtered through 0.2 µm and frozen at −80 °C until use.

### Leukemic cell isolation

Fresh and biobanked AML patient samples were obtained with informed written consent from participants at the Karolinska University Hospital, Uppsala University Hospital, and the acute leukemia biobank in compliance with the Declaration of Helsinki and approved by the regional Ethical Review Authority in Stockholm (DNR: 2017/2085-31/2, 2008/1330-31/3, 2018/2110-32). Fresh BM/PB samples were collected in culture flasks containing RPMI 1640 medium and subsequently stored/shipped at room temperature and handled within 2–72 h. MNCs were isolated via density centrifugation with Lymphoprep (STEMCELL Technologies, Vancouver, Canada) at 400 g for 20 min without brake. The MNCs were then transferred to a new vial and treated with ACK buffer (Thermo Fisher) to remove erythrocytes, after 5 min 30 mL of PBS was added, centrifugation was done at 300*g* for 5 min. Cells were first washed in PBS and finally in complete RPMI medium with 12.5% HS-5 CM before cell counting and subsequent analyses. Fresh MNCs were cryopreserved within 2 h of isolation in complete RPMI with HS-5 CM in a 1:1 ratio with FBS containing 20% DMSO and frozen in a CoolCell (Corning, Corning, NY) at a rate of −1 °C/min at −80 °C.

### Biobanked MNC thawing

Frozen MNCs were thawed in 35 mL of pre-warmed thawing buffer (RPMI 1640, 3% FBS, 8 U/mL DNase1) by heating the cryovial to 37 °C and adding the cell suspension in a slow dropwise fashion on the foam layer of a tube of thoroughly mixed buffer. Cells were incubated in the thawing buffer for 15 min at 37 °C and centrifuged at 300*g* for 7 min, after which the pellet was resuspended in 35 mL thawing buffer and incubated for 10 min at 37 °C and centrifuged again. Next, MNCs were resuspended in 1 mL complete RPMI with HS-5 CM and 50 U/mL DNase1, and incubated for 1 h (37 °C, 5% CO_2_) after which 9 mL of complete RPMI with HS-5 CM was added and incubated for an additional 2 h before use.

### Mass spectrometry-based proteomics

MNCs for proteomic analysis were washed twice in HBSS, pelleted and flash frozen. Cell pellets were dissolved in Lysis buffer (4% SDS, 50 mM HEPES pH 7.6, 1 mM DTT), heated to 95 °C and sonicated. The total protein amount was estimated with the DC protein assay (Bio-Rad, Hercules, CA). Samples were then prepared for mass spectrometry analysis using a modified version of the SP3 protein clean-up and a digestion protocol^18,19^, where proteins were digested by LysC and trypsin (sequencing grade modified, Pierce). In brief, up to 250 µg protein from each sample was alkylated with 4 mM Chloroacetamide. Sera‐Mag SP3 bead mix (20 µL, Thermo Fisher) was transferred into the protein sample together with 100% Acetonitrile to a final concentration of 70%. The mix was incubated under rotation at room temperature for 18 min. The mix was placed on the magnetic rack and the supernatant was discarded, followed by two washes with 70% ethanol and one with 100% acetonitrile. The beads-protein mixture was reconstituted in 100 µL LysC buffer (0.5 M Urea, 50 mM HEPES pH: 7.6 and 1:50 enzyme (LysC) to protein ratio) and incubated overnight. Finally, trypsin was added in 1:50 enzyme to protein ratio in 100 µL 50 mM HEPES pH 7.6 and incubated overnight. The peptides were eluted from the mixture after placing the mixture on a magnetic rack, followed by peptide concentration measurement (Bio-Rad DC Assay). The samples were then pH adjusted using TEAB pH 8.5 (100 mM final conc.), 65 µg of peptides from each sample were labeled with isobaric TMT-tags (TMT10plex reagent) according to the manufacturer’s protocol (Thermo Fisher). Each set consisted of 9 individual patient samples and the tenth channel contained the same sample pool in each set, consisting of a mixture of patient samples. Sample pools were used as denominators when calculating TMT-ratios and thus served to link the sets together. One sample pool was generated for the frozen cohort and one sample pool was generated for the fresh cohort. The sample pool from the frozen cohort was included in one of the sets of the fresh cohort. The tryptic peptides for each set were separated by immobilized pH gradient-isoelectric focusing (IPG-IEF) on both 3–10 strips and 3.7-4.9 strips as described previously^20^.

Of note, the labeling efficiency was determined by LC-MS/MS before pooling of the samples. For the sample clean-up step, a solid phase extraction (SPE strata-X-C, Phenomenex, Torrance, CA) was performed, and purified samples were dried in a SpeedVac. An aliquot of approximately 10 µg was suspended in LC mobile phase A and 1 µg was injected on the LC-MS/MS system.

Online LC-MS was performed as previously described^21^ using a Dionex UltiMate™ 3000 RSLCnano System coupled to a Q-Exactive-HF mass spectrometer (Thermo Fisher). Each of the 72 plate wells were dissolved in 20 µL solvent A and 10 µL was injected. Samples were trapped on a C18 guard-desalting column (Acclaim PepMap 100, 75 μm × 2 cm, nanoViper, C18, 5 µm, 100 Å), and separated on a 50 cm long C18 column (Easy spray PepMap RSLC, C18, 2 μm, 100 Å, 75 μm × 50 cm). The nano capillary solvent A was 95% water, 5% DMSO, 0.1% formic acid; and solvent B was 5% water, 5% DMSO, 95% acetonitrile, 0.1% formic acid. At a constant flow of 0.25 μL min−1, the curved gradient went from 6 to 8% B up to 40% B in each fraction in a dynamic range of gradient length (Supplementary Table 7), followed by a steep increase to 100% B in 5 min. FTMS master scans with 60,000 resolution (and mass range 300-1500 *m/z*) were followed by data-dependent MS/MS (30,000 resolution) on the top 5 ions using higher energy collision dissociation (HCD) at 30% normalized collision energy. Precursors were isolated with a 2 *m/z* window. Automatic gain control (AGC) targets were 1e6 for MS1 and 1e5 for MS2. Maximum injection times were 100 ms for MS1 and 100 ms for MS2. The entire duty cycle lasted ~2.5 s. Dynamic exclusion was used with 30 s duration. Precursors with unassigned charge state or charge state 1 were excluded. An underfill ratio of 1% was used.

Protein and peptide identification and quantification were carried out as previously described^21^. Briefly, Orbitrap raw MS/MS files were converted to mzML format using msConvert from the ProteoWizard tool suite^22^. Spectra were then searched using MSGF+ (v10072)^23^ and Percolator (v2.08)^24^, where search results from 8 subsequent fractions were grouped for Percolator target/decoy analysis. All searches were done against the human protein subset of Ensembl 92 in the Nextflow platform^25^. MSGF+ settings included precursor mass tolerance of 10 ppm, fully-tryptic peptides, maximum peptide length of 50 amino acids and a maximum charge of 6. Fixed modifications were TMT-10plex on lysines and peptide N-termini, and carbamidomethylation on cysteine residues, a variable modification was used for oxidation on methionine residues. Quantification of TMT-10plex reporter ions was done using OpenMS project’s IsobaricAnalyzer (v2.0)^26^. found at 1% FDR (false discovery rate) were used to infer gene identities.

Protein quantification by TMT10plex reporter ions was calculated using TMT PSM ratios to the sample pool and normalized to the sample median. The median PSM TMT reporter ratio from peptides unique to a gene symbol was used for quantification. Protein false discovery rates were calculated using the picked-FDR method using gene symbols as protein groups and limited to 1% FDR^27^.

### Drug sensitivity and resistance testing

MNCs were added to pre-spotted drug plates (FIMM HTB)^28^ or custom plates made with an Echo 550 (Beckman Coulter, Brea, CA, Supplementary Table 8). Cells were incubated in complete RPMI for 72 h (37 °C, 5% CO_2_). Cell viability was measured by CellTiterGlo (CTG, Promega, Madison, WI) on an EnSight plate reader (PerkinElmer, Waltham, MA). Drug sensitivity scores (DSS) were calculated with Breeze^29^. Selective DSS (sDSS) values were calculated by subtracting healthy BM controls DSS values from patient DSS values. Fold growth was measured in untreated cells as the ratio of luminescence signal at 0 h and 72 h.

### Flow cytometry

MNCs were stained for 30 min, 4 °C in FACS buffer (PBS, 0.5% BSA and 2 mM EDTA) before 20 min fixation in 4% PFA (Supplementary Table 9). For intracellular staining cells were fixed and permeabilized with the eBioscience Foxp3/transcription factor staining buffer set (Thermo Fisher, Waltham, MA) before incubation with intracellular antibodies. Data was acquired on a BD LSRII (BD Biosciences, Franklin Lakes, NJ) and analyzed in FlowJo (BD Biosciences) and Cytobank (Beckman Coulter).

### Statistics

Statistical analysis and visualization were performed in Prism 9 (GraphPad Software, Boston, MA) and Morpheus^30^. Data is presented as mean with standard error of the mean (SEM) unless stated otherwise. Data differences between the two main cohorts were analyzed using Welch’s t-test. The difference between timepoints were determined by the Kruskal-Wallis test. Differences in DSS between fresh and frozen cohorts were determined through multiple t-tests (FDR 1%), correlations were tested with Spearman’s rank correlation. For all paired patient data, unpaired t-tests or Wilcoxon rank test were used depending on normality. P < 0.05 was considered statistically significant. Patient characteristics differences between cohorts were tested with uni- and multivariate analysis using the compareGroups^31^ (v 4.6.0) and stats (v 4.2.2) R packages. Univariate P-values were obtained with Fisher’s exact test and Man-Whitney U test for categorical and continuous variables, respectively. Multivariate P-values were obtained using multivariate logistic regression analysis. Differential expression (DE) analysis was done on MS-proteomics data for fresh and frozen patients. The “lmFit” and “eBayes” functions from the limma^32^ (v 3.56.2) R package were used to compute the fold changes and standard errors by fitting a linear model for each protein. Gene set enrichment analysis (GSEA) was done with the clusterProfiler^33^ (v 4.8.2) R package, using a pre-ranked protein list from the DE analysis. The hallmark gene sets from the msigdbr (v 7.5.1) R package were used, excluding gene sets > 500 and < 10 and Q values were calculated using a false discovery rate of 5%. Plots were generated using the enrichplot (v 1.20.0) and ggplot2 (v 3.4.4) R packages.

### Reporting summary

Further information on research design is available in the Nature Research Reporting Summary linked to this article.

### Supplementary information

REPORTING SUMMARY

Supplemental information

## Data Availability

The data sets analyzed during the current study are not publicly available for privacy reasons but will be available from the corresponding author on reasonable request upon publication of the studies that generated the data. The MS-proteomics data will be deposited in the public repository PRIDE.
