# Supplementary material for: Delineating functional and molecular impact of ex vivo sample handling in precision medicine
Source: NPJ Precis Oncol. 2024 Feb 19;8:38. doi: 10.1038/s41698-024-00528-7 (PMC10876937; doi:10.1038/s41698-024-00528-7)
Supplement: Supplementary file 2 — Supplemental information [file 41698_2024_528_MOESM2_ESM.pdf]

Delineating functional and molecular impact of *ex vivo* sample handling in translational cancer research

Supplemental figures

Supplemental Figure 1

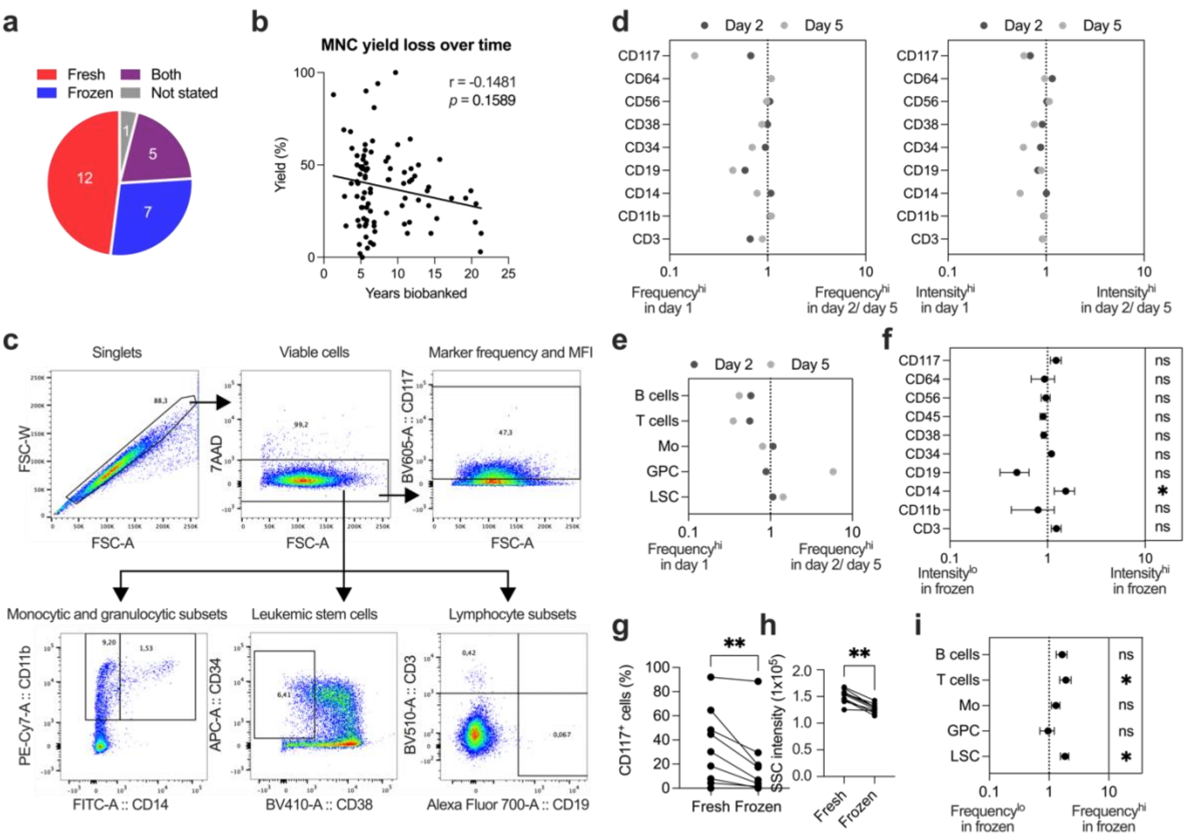

Supplemental Figure 1. (a) Summary of recent studies (n = 25) involving functional screens on hematological malignancies showing the distribution of the sample types used. (b) Comparison of cell yield, calculated as the cell count after thawing divided by cells frozen, vs. years biobanked for frozen samples (n = 92), shown with a linear regression and Spearman's rank correlation. (c) Gating strategy for cell composition flow cytometry showing frequency of parent (%), gates for each marker were based on unstained cell control and compensation beads. (d) Fold change in number of positive cells and MFI for each tested antibody (e) and for each gated population at different timepoints from sample (n = 1) acquisition to isolation. Both

are shown as the mean of two technical replicates. (f) Fold change in MFI between frozen and fresh paired samples (n = 10), shown as mean with SEM and Wilcoxon test. (g-h) CD117 frequency of live cells and SSC positive cell MFI (n = 10) before and after freezing, shown with Wilcoxon test. (i) Fold change in number of cells within each gated cell population after freezing, shown as mean with SEM and Wilcoxon test. *: $P \leq 0.05$, **: $P \leq 0.01$, ns = not significant.

Supplemental Figure 2

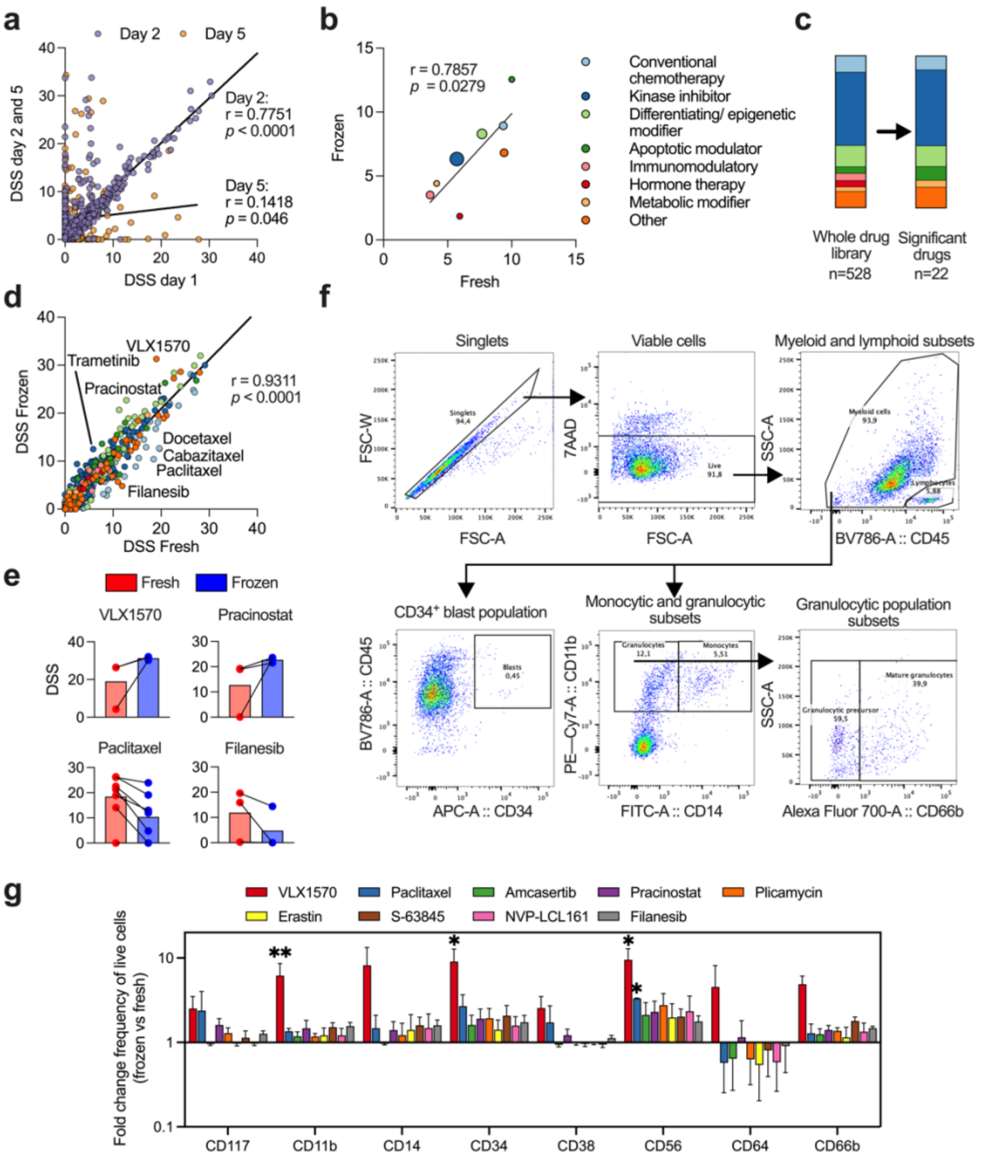

Supplemental Figure 2: (a) Correlation of drug sensitivity scores (DSS) for DSRT performed at different timepoints from sample acquisition to isolation (n = 1) shown as a linear regression and Spearman's rank correlation. (b) Correlation of drug sensitivity scores (DSS) for each drug class in the fresh (n = 107) and frozen (n = 67) cohorts, shown as linear regression and Spearman's rank correlation of average DSS score per drug class, with dot size correlating to the number of drugs in each class. (c) Drug class distribution in the whole FO5A drug library and of significantly different drugs between cohorts. (d) Drug responses between fresh and frozen paired samples (n = 7) are highly correlated here shown as a linear regression and Spearman's rank correlation of average DSS. (e) Top two drugs with the largest positive (top) or negative (bottom) dDSS for paired patients shown as mean with SEM. (f) Gating strategy

for flow cytometry-based drug screen showing frequency of parent (%), gates for each marker were based on unstained control and stained compensation beads, marker frequency was determined for all viable cells. (g) Fold change in marker frequency of viable cells between fresh and frozen after drug treatment (n = 3), IC50 for each drug is shown as mean with SEM and unpaired t tests. *: $P \leq 0.05$, **: $P \leq 0.01$, ***: $P \leq 0.001$, ns = not significant.

Supplemental Figure 3

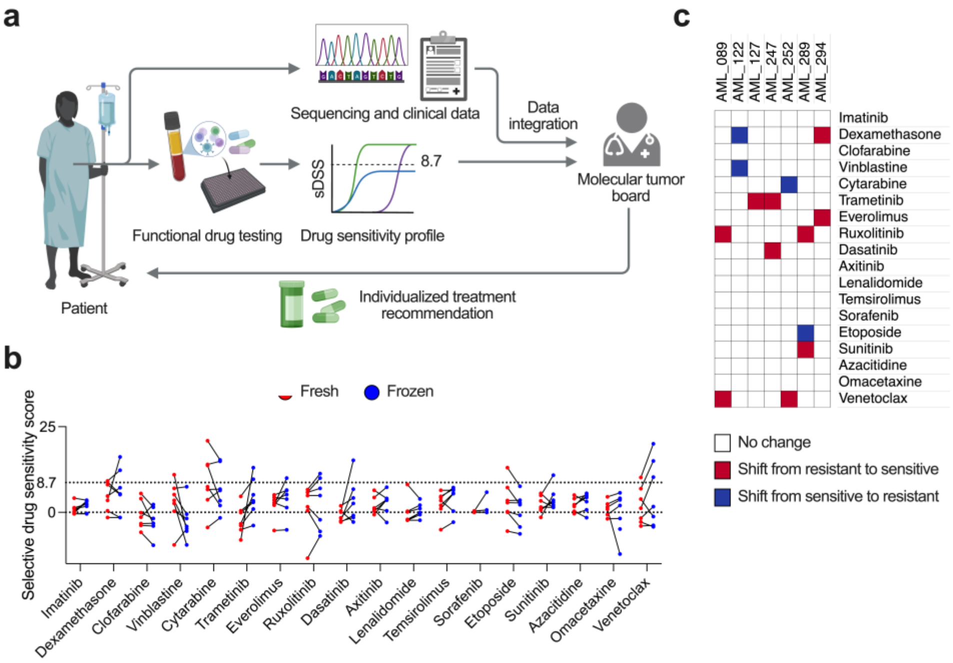

Supplemental Figure 3: (a) Simplified overview of the translational workflow in Malani et al. 2022. (b) Fresh and frozen sDSS for each paired patient (n = 7) shown for all 18 recommended drugs by the molecular tumor board. Dotted line represents the sDSS cutoff of 8.7. (c) A heatmap showing all 18 drugs recommended by the molecular tumor board. Red squares indicate drug response shifted from resistant to sensitive, blue indicates a shift from sensitive to resistant, based on the sDSS cutoff of 8.7. White indicates no change.

Supplemental tables

Supplemental Table 1: Overview of studies including functional screening assays.

Authors	Cancer type	Source	Cell type	Functional test	Sample type	Ref.
Baccelli et al 2017	AML	Not stated	MNC	Viability based *ex vivo* drug screen	Frozen	[1]
Bennett et al 2014	AML	BM	WBC	Flow cytometry-based *ex vivo* drug screen	Fresh	[2]
Bhatt et al 2020	AML	BM, PB	MNC	Dynamic BH3 profiling	Fresh	[3]
Collignon et al 2020	AML	BM, PB	MNC	Viability based *ex vivo* drug screen	Fresh	[4]
Dembitz et al 2020	AML	BM	MNC	MTT assay	Frozen	[5]
Faraoni et al 2015	AML	BM	MNC	Colorimetric based *ex vivo* drug screen	Fresh	[6]
Frismantas et al 2017	ALL	BM, PB	MNC	Flow cytometry-based *ex vivo* drug screen	Frozen	[7]
Kamens et al. 2023	ALL	PB, BM, and spleen from PDX	Leukemic cells, hCD45+	Viability based *ex vivo* drug screen	Frozen	[8]
Knorr et al 2017	AML, MDS	BM	MNC	Flow cytometry-based *ex vivo* drug screen	Fresh	[9]
Kornauth et al 2022	AML, ALL, T-NHL, B-NHL	BM, PB, LN	Not stated	Imaging based *ex vivo* drug screen	Fresh	[10]
Kurtz et al 2017	AML, CLL	BM, PB	MNC	Colorimetric based *ex vivo* drug screen	Fresh	[11]
Kuusanmäki et al 2020	AML	BM	MNC	Viability and flow cytometry-based *ex vivo* drug screen	Both	[12]
Lamble et al 2020	AML	BM, PB	MNC	T-cell proliferation assay	Fresh	[13]
Leung et al 2019	AML	BM, PB	MNC	Flow cytometry-based *ex vivo* drug screen	Frozen	[14]
Lin et al 2020	AML	BM	WBM	Flow cytometry-based *ex vivo* drug screen	Fresh	[15]
Malani et al 2022	AML	BM, PB	MNC	Viability based *ex vivo* drug screen	Fresh	[16]
Metts et al 2017	AML	CB, BM	MNC	Flow cytometry-based *ex vivo* drug screen	Both	[17]
Onecha et al 2020	AML	BM	WBC	Flow cytometry-based *ex vivo* drug screen	Fresh	[18]
Pan et al 2014	AML	PB. BM	MNC	Flow cytometry-based *ex vivo* drug screen, dynamic BH3 profiling	Both	[19]
Pei et al 2020	AML	BM, PB	ROS-low LSCs	Flow cytometry-based *ex vivo* drug screen	Frozen	[20]
Ramsey et al 2020	AML	BM	MNC	Viability and flow cytometry-based *ex vivo* drug screen	Not stated	[21]

Simon et al 2017	AML	Not stated	MNC	Viability based *ex vivo* drug screen	Frozen	[22]
Spinner et al 2020	AML, MDS, MDS/MPN	BM, PB	WBC	Flow cytometry-based *ex vivo* drug screen	Fresh	[23]
Swords et al 2017	AML	BM, PB	MNC	Viability based *ex vivo* drug screen	Both	[24]
Tavor et al 2020	AML	BM, PB	MNC	Viability based *ex vivo* drug screen	Frozen	[25]
Tyner et al 2018	AML	BM, PB	MNC	Colorimetric based *ex vivo* drug screen	Fresh	[26]

ALL indicates acute lymphoblastic leukemia; AML, acute myeloid leukemia; B-NHL, B cell non-Hodgkin's lymphoma; CB, cord blood; CLL, chronic lymphocytic leukemia; LN, lymph node; MDS, myelodysplastic syndromes; MDS/MPN, myelodysplastic syndrome/myeloproliferative neoplasm; PDX, patient derived xenograft; ROS, reactive oxygen species; T-NHL, T cell non-Hodgkin's lymphoma; and WBC, white blood cells.

Supplemental Table 2: Clinical and cytogenetic characteristics comparison for fresh and frozen cohorts

Clinical and cytogenetic characteristics for DSRT and MS-based proteomic samples (n, %, unless specifically stated)								
	DSRT samples				MS-based proteomics samples			
	Frozen cohort (n = 67)	Fresh cohort (n = 107)	P-value (univariate)	P-value (multivariate)	Frozen cohort (n = 118)	Fresh cohort (n = 43*)	P-value (univariate)	P-value (multivariate)
**Women**	31 (46.3)	42 (39.3)	0.45	0.99	63 (53.4)	20 (46.5)	0.55	0.56
**Age (median, range)**	66 (29 - 84)	62 (23 - 89)	0.70	0.69	63 (23 - 84)	67 (23 - 89)	0.06	0.007
**AHD-AML****	13 (19.4)	9 (8.4)	0.06	0.03	23 (19.5)	4 (9.3)	0.20	0.07
**Relapse sample**	0 (0)	9 (8.4)	0.01	0.99	0 (0)	3 (7.0)	0.02	0.99
**Bone marrow blasts (median, range,)**	49.5 (11 - 100)	50 (0 - 98)	0.91	0.72	55 (14 - 100)	55 (26.8 - 98)	0.42	0.58
**WBC counts (median range, per mm^3)**	42.7 (0.6 - 296.5)	23.8 (0.2 - 473.6)	0.03	0.87	26.6 (0.5 - 296.5)	37.4 (1 - 473.6)	0.38	0.05
**Cytogenetic aberrations**								
t(15;17)	0 (0)	2 (1.9)	0.52	0.99	3 (2.5)	0 (0)	0.57	0.99
CBF-AML***	6 (9.0)	8 (7.5)	0.95	0.24	9 (7.6)	5 (11.6)	0.53	0.16
Normal karyotype	36 (53.7)	44 (41.1)	0.14	0.08	59 (50.0)	15 (34.9)	0.13	0.40
Complex karyotype	7 (10.4)	16 (15.0)	0.53	0.30	8 (6.8)	7 (16.3)	0.12	0.04
del(5q)/-5 or del(7q)/-7	10 (14.9)	14 (13.1)	0.91	0.14	11 (9.3)	6 (14.0)	0.40	0.21
Other****	2 (3.0)	1 (0.9)	0.56	0.99	3 (2.5)	1 (2.3)	1	0.99

Footnotes: P-values for univariate analyses were obtained by applying Fisher's exact test for categorial variables and Mann-Whitney U test for continuous variables. Multivariate P-values were obtained by multivariate logistic regression analysis including all variables. *Actual number of samples n=44 including one patient at diagnosis and relapse; **AHD-AML includes both therapy-related AML and secondary AML. ***CBF-AML includes t(8;21), inv(16) and t(16;16); ****Other cytogenetic aberrations includes inv(3), t(3;3) and 11q23 abnormalities. Abbreviations: AHD-AML: AML with an antecedent hematological disease; CBF-AML: Core binding factor AML; WBC: White blood cell.

Supplemental Table 3: Mutational characteristics comparison for fresh and frozen cohorts

Mutational characteristics for DSRT and MS-based proteomics samples (n, %)								
	DSRT samples				MS-based proteomics samples			
	Frozen cohort (n = 67)	Fresh cohort (n = 107)	P-value (univariate)	P-value (multivariate)	Frozen cohort (n = 118)	Fresh cohort (n = 43*)	P-value (univariate)	P-value (multivariate)
ASXL1	8 (11.9)	9 (8.4)	0.62	0.26	15 (12.7)	4 (9.3)	0.75	0.96
***CEBPA* (single)**	6 (9.0)	6 (5.6)	0.54	0.23	11 (9.3)	2 (4.7)	0.52	0.36
***CEBPA* (double)**	3 (4.5)	0 (0)	0.06	0.99	8 (6.8)	0 (0)	0.11	0.99
DNMT3A	17 (25.4)	21 (19.6)	0.48	0.97	21 (17.8)	11 (25.6)	0.38	0.29
EZH2	3 (4.5)	3 (2.8)	0.68	0.45	2 (1.7)	2 (4.7)	0.29	0.24
FLT3**	9 (13.4)	29 (27.1)	0.33	0.67	41 (34.7)	11 (25.6)	0.36	0.19
IDH1	3 (4.5)	5 (4.7)	1	0.48	8 (6.8)	4 (9.3)	0.74	0.46
IDH2	16 (23.9)	14 (13.1)	0.10	0.04	28 (23.7)	11 (25.6)	0.97	0.84
KIT	2 (3.0)	4 (3.7)	1	0.34	7 (5.9)	1 (2.3)	0.68	0.19
KRAS	2 (3.0)	4 (3.7)	1	0.71	2 (1.7)	2 (4.7)	0.29	0.41
NPM1	27 (40.3)	29 (27.1)	0.10	0.03	38 (32.2)	14 (32.6)	1	0.81
NRAS	6 (9.0)	14 (13.1)	0.56	0.21	11 (9.3)	9 (20.9)	0.09	0.01
PHF6	0 (0.00)	2 (1.9)	0.52	0.99	3 (2.5)	1 (2.3)	1	0.99
PTPN11	2 (3.0)	4 (3.7)	1	0.33	6 (5.1)	2 (4.7)	1	0.63
RAD21	2 (3.0)	4 (3.7)	1	0.55	1 (0.9)	2 (4.7)	0.17	0.19
RUNX1	6 (9.0)	12 (11.2)	0.83	0.87	18 (15.3)	6 (14.0)	1	0.92
SF3B1	1 (1.5)	2 (1.9)	1	0.93	4 (3.4)	0 (0)	0.57	0.99
SMC1A	2 (3.0)	3 (2.8)	1	0.95	1 (0.9)	0 (0)	1	0.99
SMC3	1 (1.5)	1 (0.9)	1	0.99	2 (1.7)	0 (0)	1	0.99
STAG2	6 (9.0)	6 (5.6)	0.54	0.13	9 (7.6)	2 (4.7)	0.73	0.12
TET2	15 (22.4)	20 (18.7)	0.69	0.92	22 (18.6)	8 (18.6)	1	0.72
TP53	6 (9.0)	6 (5.6)	0.54	0.04	6 (5.1)	3 (7.0)	0.70	0.90
U2AF1	1 (1.5)	1 (0.9)	1	0.23	3 (2.5)	0 (0)	0.57	0.99
WT1	3 (4.5)	11 (10.3)	0.28	0.11	6 (5.1)	1 (2.3)	0.68	0.65

Footnotes: P-values for univariate analyses were obtained by applying Fisher's exact test whereas multivariate P-values were obtained by multivariate logistic regression analysis including all genes. *Actual number of samples n=44 including one patient at diagnosis and relapse. **FLT3 denotes both tyrosine kinase domain point mutations and internal tandem duplications.

Supplemental Table 4: Multivariate logistic regression analyses of fresh and frozen cohorts in both DSRT and MS-proteomic datasets.

Multivariate logistic regression analyses for DSRT and MS-based proteomics samples						
	DSRT samples			MS-based proteomics samples		
	Odds ratio	95 % CI	P-value	Odds ratio	95 % CI	P-value
**Gender (men vs women)**	0.97	0.49 - 1.94	0.93	0.71	0.33 - 1.51	0.37
**Age (continuous)**	1.00	0.98 - 1.03	0.70	0.97	0.94 - 0.99	0.01
**AHD-AML* (yes vs no)**	3.29	1.25 - 9.01	0.02	3.36	1.11 - 12.87	0.04
**Normal karyotype (yes vs no)**	1.46	0.64 - 3.33	0.36	2.20	0.96 - 5.19	0.07
**Complex karyotype (yes vs no)**	–	–	–	0.47	0.13 - 1.62	0.23
**White blood cells (continuous)**	1.00	0.99 - 1.01	0.69	–	–	–
**Molecular aberration (yes vs no)**						
IDH2	1.95	0.82 - 4.64	0.13	–	–	–
NPM1	1.26	0.52 - 3.03	0.61	–	–	–
NRAS	–	–	–	0.32	0.11 - 0.91	0.03
RAD21	–	–	–	0.07	0.003 - 0.89	0.04

Footnotes: Variables with P < 0.2 in univariate analyses that had at least one observation per condition were included in addition to correction for age and sex. *AHD-AML includes both therapy-related AML and secondary AML. **FLT3 denotes both tyrosine kinase domain point mutations and internal tandem duplications. Abbreviations: CI: Confidence interval; AHD-AML: Acute myeloid leukemia with an antecedent hematological disease.

Supplemental Table 5: Proteins with significantly different log2 fold change between both cohorts.

Proteins	logFC	AveExpr	t	p_value	q_value	B	log10p	log10q
**THAP4**	0.79963889	0.22721779	9.18332398	2.68E-16	1.29E-12	26.3565259	15.5715611	11.8882888
**SYS1**	-0.8385379	-0.0425371	-9.5123226	1.51E-16	1.29E-12	25.266547	15.8206609	11.8882888
**PLEKHG2**	1.1044173	0.08527405	9.620742	5.27E-16	1.69E-12	24.0047769	15.2782034	11.7710224
**ZNF397**	0.89186274	0.10003306	8.38018385	8.21E-14	1.98E-10	19.7223029	13.085624	9.70338179
**ZBTB38**	0.82024109	0.20871813	7.79569413	1.50E-12	2.90E-09	17.8174275	11.8229742	8.53764201
**IGF2BP1**	2.1164395	-1.1536929	7.49053678	1.33E-11	1.61E-08	15.9524595	10.8750041	7.7937919
**ZNF667**	1.31122507	0.10477972	7.57568504	1.21E-11	1.61E-08	15.2628688	10.9189108	7.7937919
**C17orf53**	1.12936309	-0.0552553	7.64262028	1.28E-11	1.61E-08	15.0139539	10.8941448	7.7937919
**TUSC2**	0.75882612	0.14356341	7.03118598	8.69E-11	9.32E-08	14.1829596	10.0607851	7.03072534
**ETNK1**	-0.418305	-0.0681392	-6.890828	1.15E-10	1.01E-07	13.9334515	9.93743056	6.99452101
**CALML5**	-1.4949292	-0.0192112	-7.1200769	1.14E-10	1.01E-07	13.9224322	9.94175972	6.99452101
**FAM122B**	0.40885501	0.03600834	6.68536044	3.50E-10	2.82E-07	12.8685427	9.45563662	6.55051563
**BTBD8**	0.51898391	0.04441194	6.55501103	9.15E-10	6.79E-07	11.9450451	9.03871258	6.1683537
**ASS1**	1.12049022	-0.8631635	6.40208416	1.57E-09	1.08E-06	11.4302497	8.8042167	5.9660425
**GSTM5**	1.18821903	-0.9449099	6.41628107	1.87E-09	1.13E-06	11.2705981	8.72913438	5.94661432
**HBG1**	1.74363743	-1.1412777	6.41522764	1.88E-09	1.13E-06	11.2655063	8.72679657	5.94661432
**MAP1B**	1.0678421	-0.03661	6.21323904	5.94E-09	3.37E-06	10.1802038	8.22588572	5.47203241
**MRPL34**	-0.5594139	-0.1022795	-6.0849185	8.06E-09	4.32E-06	9.86381124	8.09365078	5.36462106
**MT-ATP6**	-0.5407493	-0.0138554	-6.0237738	1.10E-08	5.58E-06	9.56744498	7.95906147	5.25351284
**MYO10**	0.56447095	0.08870409	6.01675292	1.39E-08	6.70E-06	9.37307351	7.85713764	5.17386541
**PDCD2L**	0.35693829	0.07556607	5.79947725	3.37E-08	1.51E-05	8.49656476	7.47227531	4.81969904
**TMEM258**	-0.2788395	0.01558963	-5.7944904	3.45E-08	1.51E-05	8.47305236	7.46157859	4.81969904
**FUNDC1**	-0.3470738	0.02878613	-5.7663452	3.97E-08	1.66E-05	8.34059914	7.4013129	4.7787385
**KIAA1549**	1.40739717	0.20898706	6.01959494	4.33E-08	1.74E-05	7.99226833	7.36365844	4.75956744
**MT-ND1**	-0.4890684	0.01203568	-5.6604015	7.20E-08	2.78E-05	7.80202414	7.14273989	4.55637767
**TGFBR1**	0.31345103	0.11238955	5.62567929	8.51E-08	3.04E-05	7.64342562	7.07017558	4.51723712
**MT-ND5**	-0.3143036	0.09217672	-5.6127998	8.40E-08	3.04E-05	7.62548402	7.07570842	4.51723712
**CDKN2D**	0.57069695	-0.0098691	5.59205882	1.00E-07	3.44E-05	7.49046338	7.00017115	4.46302694
**RASAL1**	1.56455471	-0.6001004	5.66793357	1.33E-07	4.01E-05	7.27902619	6.8759407	4.39678845
**MMACHC**	0.49939117	-0.0420831	5.55189431	1.31E-07	4.01E-05	7.25681259	6.88226321	4.39678845
**SLC25A6**	-0.2588828	0.08885993	-5.5271755	1.27E-07	4.01E-05	7.23229769	6.89650732	4.39678845
**UCHL1**	-1.0600512	-0.5675619	-5.5193084	1.32E-07	4.01E-05	7.19637608	6.88012883	4.39678845
**COL14A1**	1.24246525	-0.1900386	5.50471071	1.64E-07	4.79E-05	7.04795072	6.78587512	4.32008683
**NEK5**	-0.9120469	-0.0469355	-5.5815344	1.69E-07	4.79E-05	6.80331016	6.77240793	4.31958461
**C1orf167**	0.42530956	0.14503782	5.51588015	2.03E-07	5.30E-05	6.85328721	6.69156336	4.27546285
**ZBED8**	-0.3221418	-0.0333401	-5.4463539	2.00E-07	5.30E-05	6.83452878	6.69975472	4.27546285
**CASP14**	-0.5293427	-0.0258479	-5.4313455	2.00E-07	5.30E-05	6.79710916	6.69800463	4.27546285
**BDP1**	-0.3516008	-0.1027862	-5.4365714	2.25E-07	5.71E-05	6.74834999	6.64754801	4.24302938
**HERC6**	0.5911483	-0.0662069	5.4287016	2.49E-07	6.00E-05	6.65814923	6.60415423	4.22191199
**ADCK5**	-0.3823293	0.00498194	-5.4027068	2.45E-07	6.00E-05	6.64028062	6.61071623	4.22191199
**CCDC71**	-0.6618749	0.03081128	-5.4253045	3.10E-07	7.29E-05	6.26557238	6.50878346	4.13726508
**NES**	0.77910473	-0.1362951	5.34238439	3.25E-07	7.46E-05	6.37355256	6.48839657	4.12734363
**RSAD2**	1.05139014	-1.1559614	5.33503394	3.60E-07	8.08E-05	6.30641244	6.4433577	4.09252392
**LRRC32**	1.50189003	-0.310704	5.34938337	3.98E-07	8.73E-05	5.98140143	6.39974578	4.05889623
**NOL3**	0.73839041	0.11505877	5.32886595	4.32E-07	9.27E-05	6.14361183	6.36407216	4.03298244
**MT-CO1**	-0.6814628	-0.0703816	-5.2522573	4.65E-07	9.74E-05	5.99793153	6.33300149	4.01145709
**TMCO4**	0.30677106	0.05055755	5.23688539	4.99E-07	0.00010237	5.93020796	6.30204023	3.98983586
**RC3H1**	0.21687405	0.04852998	5.23969865	5.22E-07	0.00010493	5.92419826	6.28216518	3.97910419
**SMURF2**	0.3164756	0.08335906	5.2136964	5.88E-07	0.00011581	5.81136242	6.23034587	3.93623972
**CD99**	-0.517542	-0.0506155	-5.1852867	6.33E-07	0.00012212	5.70390617	6.19854506	3.91321283
**USP18**	1.22988623	-0.3721604	5.25604948	7.22E-07	0.00013657	5.67464489	6.14138674	3.86465469

**PBXIP1**	-0.5311892	-0.092552	-5.1291546	8.19E-07	0.00015191	5.45953109	6.08671923	3.81842034
**SDHC**	-0.444115	-0.0737793	-5.1303913	8.55E-07	0.00015553	5.42796868	6.06820055	3.80817418
**TTC9C**	0.33605523	-0.0627644	5.11230104	8.85E-07	0.00015798	5.38652879	6.05329988	3.80139141
**COX11**	-0.3252385	0.09819525	-5.1017774	9.79E-07	0.00017169	5.33015102	6.00919237	3.76525283
**SLC25A22**	-0.3076773	0.04234529	-5.0795306	1.03E-06	0.00017684	5.24507519	5.98852635	3.75241215
**KCNQ5**	-0.448441	-0.0666602	-5.1228692	1.09E-06	0.00018469	5.28425046	5.96198358	3.7335562
**BTBD7**	0.35889828	0.0740135	5.07239541	1.19E-06	0.00019049	5.18916841	5.92628065	3.72012967
**GGT7**	-0.536131	-0.0432528	-5.0636345	1.16E-06	0.00019049	5.16782262	5.93452901	3.72012967
**RPS29**	0.80134769	-0.3383517	5.0585556	1.18E-06	0.00019049	5.14738466	5.92724315	3.72012967
**HPGD**	1.12805539	-0.4208045	5.04252218	1.21E-06	0.00019198	5.08611651	5.91570768	3.71673529
**CSAD**	0.73834141	0.0831107	5.09059254	1.35E-06	0.00021075	5.08670489	5.86814297	3.67623243
**ABHD3**	-0.6471719	-0.003726	-5.0499901	1.48E-06	0.000226	5.00669075	5.8308494	3.64588771
**DMAC2**	-0.3424347	0.02111901	-4.9171025	2.23E-06	0.00033652	4.55227904	5.65110572	3.47298346
**USP30**	0.36532242	0.12654515	4.9175364	2.35E-06	0.00033812	4.54858304	5.62915809	3.47093067
**ACD**	-0.2275169	-0.0035395	-4.9087112	2.32E-06	0.00033812	4.51742184	5.63504086	3.47093067
**MYC**	-0.5940475	-0.254114	-4.8993505	2.30E-06	0.00033812	4.47914352	5.63735278	3.47093067
**DRAM2**	-0.3004672	-0.0286344	-4.8854782	2.45E-06	0.00034365	4.42101421	5.61066858	3.46388022
**SLC25A11**	-0.3185697	0.0511549	-4.8847834	2.46E-06	0.00034365	4.41810584	5.60933336	3.46388022
**DLAT**	-0.1921745	0.03939813	-4.8516835	2.85E-06	0.00039204	4.27991353	5.54587626	3.40667207
**FARP2**	-0.5012643	-0.0527871	-4.8791869	2.92E-06	0.0003967	4.37190231	5.53458202	3.40153814
**E4F1**	-0.2745324	0.01669254	-4.8453277	3.06E-06	0.00039883	4.25552087	5.51428214	3.39921162
**PPTC7**	-0.3031672	0.00431672	-4.8407103	2.99E-06	0.00039883	4.23425401	5.52490356	3.39921162
**ASTE1**	-0.1839483	-0.0265253	-4.8365969	3.04E-06	0.00039883	4.21715793	5.51705006	3.39921162
**CDKN2A**	1.06421555	-0.2098998	4.91772758	3.37E-06	0.00043352	4.26416216	5.47223338	3.36299241
**RTKN**	0.57360768	0.07068808	4.83327152	4.02E-06	0.0005107	4.08677535	5.39532365	3.29183501
**FUNDC2**	-0.1784097	0.10877282	-4.7676529	4.11E-06	0.00051489	3.93222641	5.38609487	3.28828336
**TOR3A**	-0.2067577	0.05402722	-4.7513177	4.41E-06	0.0005457	3.86516455	5.35525484	3.26304721
**SCIN**	0.89812289	-0.1626849	4.80755076	4.84E-06	0.00059126	3.92292531	5.31489547	3.22822033
**SOX11**	-0.581194	0.16501192	-4.7303388	5.52E-06	0.00066514	3.7851445	5.25829919	3.17708695
**SLC25A44**	-0.2709139	-0.005046	-4.6778162	6.31E-06	0.00075096	3.57538512	5.20019967	3.12438246
**TRIP6**	0.38301388	-0.0139633	4.66838272	6.57E-06	0.00077224	3.53760962	5.18273341	3.11224503
**B3GALNT1**	-0.4029976	0.02020265	-4.6592987	6.82E-06	0.00079303	3.50128729	5.16593678	3.10071264
**NXN**	0.89681014	0.12695262	4.68194871	7.15E-06	0.00082089	3.55196029	5.14573653	3.08571359
**DHX58**	0.32774807	-0.0330617	4.61418924	7.96E-06	0.00090291	3.30906985	5.09923687	3.04435356
**YAF2**	-0.7176707	-0.0854288	-4.6533274	8.05E-06	0.00090304	3.44421033	5.09409468	3.04429089
**PPP3R1**	-0.4458909	0.1350045	-4.6052468	8.90E-06	0.00098704	3.25974192	5.05044659	3.00566361
**ATF6**	-0.3273987	-0.0194249	-4.5984141	9.21E-06	0.00100905	3.27328672	5.03590625	2.99608669
**S100A7**	-0.6897049	0.14297514	-4.5954412	9.66E-06	0.00102681	3.1589204	5.01503248	2.98850811
**DIP2A**	0.19110901	0.08166179	4.5695892	9.61E-06	0.00102681	3.13087811	5.0170867	2.98850811
**PSAT1**	0.57924176	-0.2930263	4.56778162	9.69E-06	0.00102681	3.12368422	5.01376895	2.98850811
**RAC3**	0.92064845	-0.7075129	4.57596663	1.01E-05	0.00102847	3.1859275	4.99517088	2.98780907
**AKR1C1**	-0.7308221	0.02308788	-4.6622406	1.03E-05	0.00102847	3.16534763	4.98533957	2.98780907
**MR1**	-0.2118468	-0.0203268	-4.5621205	1.03E-05	0.00102847	3.11604091	4.98764468	2.98780907
**USP38**	-0.254756	0.01163503	-4.5617283	9.94E-06	0.00102847	3.09960881	5.00266492	2.98780907
**COX6C**	-0.2200652	0.01966068	-4.5546807	1.02E-05	0.00102847	3.0716098	4.9897499	2.98780907
**MAML3**	0.276414	-0.005031	4.55410869	1.03E-05	0.00102847	3.06933875	4.98870227	2.98780907
**EIF6**	0.14373896	0.06444536	4.54959833	1.05E-05	0.00102951	3.05143899	4.98044487	2.98736871
**SLC35B3**	-0.378635	-0.0839507	-4.5506873	1.08E-05	0.00105155	3.07111806	4.96683696	2.97816993
**RPL35**	0.19486439	-0.0069131	4.53921587	1.09E-05	0.00105401	3.01028714	4.96145866	2.97715643
**RAF1**	-0.1736742	-0.0212037	-4.5227092	1.17E-05	0.00110856	2.94501064	4.93133552	2.95523884
**ALG12**	-0.2377246	0.01815789	-4.5224927	1.17E-05	0.00110856	2.94415562	4.9309409	2.95523884
**SLC25A5**	-0.2465285	0.00932092	-4.5178147	1.20E-05	0.00111956	2.92569042	4.92241826	2.95095325
**C17orf97**	0.6734266	0.12952326	4.53830694	1.35E-05	0.00121178	2.97283106	4.8698904	2.91657551
**CCDC171**	0.39498195	0.15481646	4.52164921	1.32E-05	0.00121178	2.94132781	4.88033172	2.91657551
**MTERF1**	-0.2400193	0.03193246	-4.499317	1.34E-05	0.00121178	2.87033037	4.87378807	2.91657551
**SLC4A7**	-0.4415372	-0.106384	-4.488724	1.35E-05	0.00121178	2.81119287	4.869557	2.91657551
**ADI1**	0.21952701	-0.0467634	4.48756396	1.36E-05	0.00121178	2.80663888	4.86745399	2.91657551
**NDUFA6**	-0.2012998	0.07353425	-4.481488	1.39E-05	0.00123149	2.78280166	4.85644537	2.90956964
**UBE2A**	0.21259515	-0.0084946	4.46782234	1.47E-05	0.00129177	2.72928017	4.83172373	2.88881418
**PTPN14**	-0.4633635	-0.0368198	-4.468011	1.58E-05	0.00136202	2.72289294	4.80113963	2.86581646
**ASNS**	0.430792	-0.0588819	4.45074281	1.58E-05	0.00136202	2.66256677	4.80090067	2.86581646
**PABPC1**	0.14001118	0.02559314	4.44780883	1.60E-05	0.0013665	2.65112652	4.79561412	2.86439033
**DEPDC5**	0.1344228	0.06135274	4.4395528	1.66E-05	0.00139336	2.61896587	4.78075122	2.85593545
**IFIT2**	0.50669824	-0.4376246	4.43887943	1.66E-05	0.00139336	2.61634488	4.77953985	2.85593545
**AAED1**	0.26719817	0.03142123	4.44149887	1.70E-05	0.00140905	2.64641881	4.76993042	2.85107324

**BP2**	-0.3672779	-0.003309	-4.4320102	1.71E-05	0.00140905	2.58962499	4.76718961	2.85107324
**IFI44L**	0.69948834	-0.4244937	4.45281666	1.74E-05	0.00141971	2.64269615	4.76022118	2.84780096
**MRPL3**	-0.1972139	0.00841443	-4.4215679	1.78E-05	0.00144649	2.5490684	4.74844107	2.8396858
**SMAD5**	-0.3069745	-0.0539214	-4.3774487	2.14E-05	0.0017086	2.3785415	4.66957186	2.76736062
**COX7B**	-0.3519731	-0.0190581	-4.3770588	2.14E-05	0.0017086	2.37704074	4.66887748	2.76736062
**TMEM62**	-0.319736	0.00538056	-4.3736626	2.17E-05	0.00171835	2.36397022	4.66282972	2.76488732
**SERAC1**	-0.3472257	-0.0018771	-4.3689634	2.22E-05	0.00172452	2.34589835	4.65446721	2.76333242
**SLC25A3**	-0.2259154	0.04190808	-4.3688205	2.22E-05	0.00172452	2.34534894	4.65421297	2.76333242
**PLPP1**	-0.9814868	0.03218833	-4.409389	2.28E-05	0.00174567	2.49369385	4.64272994	2.75803821
**TMEM101**	-0.3972568	0.15220421	-4.3949725	2.29E-05	0.00174567	2.43326537	4.63944681	2.75803821
**STAMBPL1**	-0.2996479	0.00348112	-4.3600015	2.30E-05	0.00174567	2.31147591	4.63853672	2.75803821
**NKIRAS2**	0.16660734	0.07773618	4.35608433	2.34E-05	0.00176	2.29644732	4.63158077	2.75448851
**NDUFAF6**	-0.2869176	0.04232659	-4.3515759	2.38E-05	0.00177882	2.27916367	4.62358045	2.74986793
**MRPS18C**	-0.4013743	0.06317924	-4.3636887	2.40E-05	0.0017793	2.29146304	4.62010937	2.74975049
**MAP2**	0.61149143	0.32405659	4.46384142	2.44E-05	0.00179294	2.46637514	4.6134645	2.74643356
**CRY1**	-0.8962253	-0.019474	-4.4391375	2.49E-05	0.00181737	2.3661831	4.6042849	2.7405566
**ZNF296**	-0.8888292	-0.1397108	-4.3840072	2.53E-05	0.00183182	2.36943695	4.59649414	2.73711714
**CTPS1**	0.17939809	-0.0056742	4.33505543	2.54E-05	0.00183182	2.21595071	4.59431457	2.73711714
**ZMYM5**	0.36107529	0.11831685	4.39026145	2.58E-05	0.00183394	2.38496782	4.58809555	2.73661387
**AKIP1**	-0.3893943	-0.0206315	-4.3469939	2.59E-05	0.00183394	2.31311452	4.58737719	2.73661387
**SMYD4**	0.22332831	0.02629805	4.33831554	2.68E-05	0.0018853	2.28069952	4.57220199	2.72462032
**KMT5C**	-0.2717973	0.0056134	-4.3201222	2.70E-05	0.00189014	2.15897413	4.56792828	2.72350514
**SWI5**	0.18934238	0.05106888	4.32879321	2.77E-05	0.00191142	2.19684273	4.5572778	2.71864387
**CTDNEP1**	-0.1713241	0.00886613	-4.3138234	2.77E-05	0.00191142	2.13498831	4.55681807	2.71864387
**ZBTB41**	-0.550118	0.06858263	-4.332904	2.95E-05	0.00201895	2.25446629	4.52995841	2.69487529
**VDAC3**	-0.2074244	0.05107163	-4.2899154	3.06E-05	0.00207581	2.04419769	4.51475194	2.6828123
**MECR**	-0.2338966	0.00285415	-4.2877868	3.08E-05	0.00207581	2.03613405	4.51101488	2.6828123
**CFAP36**	0.3138052	0.0901026	4.28649763	3.10E-05	0.00207581	2.03125155	4.50875204	2.6828123
**TMTC3**	-0.2765976	0.03117816	-4.2813718	3.16E-05	0.00210282	2.01185059	4.49975989	2.67719705
**EXTL3**	-0.2432322	-0.0017724	-4.2798812	3.18E-05	0.00210282	2.00621225	4.49714642	2.67719705
**FASN**	0.22288528	0.00279687	4.27053204	3.31E-05	0.00216878	1.97088324	4.48076906	2.66378416
**CBR4**	-0.3061192	0.01879436	-4.2688303	3.33E-05	0.00216895	1.96445912	4.47779073	2.66375021
**ZSWIM3**	-0.3300111	-0.064286	-4.2894634	3.36E-05	0.00217743	2.12437882	4.47317227	2.66205631
**OSTC**	-0.3029037	0.0235741	-4.2627227	3.41E-05	0.00219332	1.94142005	4.46710863	2.65889766
**TXNDC15**	-0.2954743	0.02274835	-4.2645558	3.48E-05	0.00222546	1.97507858	4.45790452	2.65257924
**BCL9**	1.06592804	0.10064722	4.29979151	3.52E-05	0.0022365	2.07853969	4.45289071	2.65043206
**DMPK**	0.35255697	-0.0235528	4.36162034	3.62E-05	0.0022642	2.04397782	4.44186611	2.6450846
**PDP2**	-0.4738982	-0.0055526	-4.2818204	3.61E-05	0.0022642	2.01915438	4.44246008	2.6450846
**MYB**	-0.4316362	-0.1973695	-4.2451637	3.66E-05	0.00227778	1.87532997	4.43645881	2.64248827
**CSNK1G1**	-0.2523052	-0.0621183	-4.2290191	3.91E-05	0.00241446	1.81475522	4.40835747	2.61717983
**NDUFB11**	-0.1605617	0.07721448	-4.2189074	4.07E-05	0.00249808	1.77690934	4.39079574	2.60239316
**SLC15A3**	-0.6193209	-0.2439318	-4.2193093	4.17E-05	0.00250487	1.80682834	4.37954301	2.60121399
**UQCC2**	-0.2026154	0.01108959	-4.2140955	4.15E-05	0.00250487	1.75892532	4.38244933	2.60121399
**PET117**	-0.2623216	0.0319255	-4.2138011	4.15E-05	0.00250487	1.75782552	4.38193888	2.60121399
**SMPD4**	-0.1172863	0.03062953	-4.2119272	4.18E-05	0.00250487	1.75082642	4.37869035	2.60121399
**PEX11B**	-0.1948472	0.04481914	-4.2048429	4.30E-05	0.00256075	1.72439011	4.36641921	2.59163199
**CCDC28A**	-0.2493541	-0.0974634	-4.1996925	4.51E-05	0.00263082	1.73432145	4.34575189	2.57990862
**HSDL1**	-0.2885582	-0.0186077	-4.1938641	4.49E-05	0.00263082	1.68349037	4.347431	2.57990862
**TMEM164**	-0.558532	-0.0781976	-4.1937268	4.50E-05	0.00263082	1.68297964	4.34719386	2.57990862
**NFE2**	-0.32461	-0.0827788	-4.1919376	4.53E-05	0.00263082	1.67632243	4.34410276	2.57990862
**RAB22A**	-0.1370081	0.05232835	-4.1874385	4.61E-05	0.00266227	1.65959295	4.33633437	2.57474861
**TUBA8**	0.56193963	-0.0741876	4.17774351	4.79E-05	0.00275029	1.62359142	4.31961452	2.56062156
**TMEM259**	-0.1815146	0.04028268	-4.1711202	4.92E-05	0.00280677	1.59903499	4.30820809	2.55179256
**ROMO1**	-0.459018	0.01020968	-4.180628	5.01E-05	0.00284275	1.70038993	4.30011462	2.54626131
**KLF2**	-0.7890637	-0.0503785	-4.1923715	5.10E-05	0.00286324	1.64994546	4.29282701	2.54314176
**PCBP2**	-0.1076752	0.06852146	-4.1616467	5.11E-05	0.00286324	1.56396527	4.29191554	2.54314176
**REXO1**	0.18312372	0.03063051	4.16592916	5.31E-05	0.00295611	1.64714336	4.27510773	2.52927938
**ZNF496**	0.34843022	0.08854736	4.17654178	5.39E-05	0.00295611	1.59551468	4.26806894	2.52927938
**RDH11**	-0.2829595	0.01606612	-4.1496954	5.35E-05	0.00295611	1.51981494	4.27139979	2.52927938
**CC2**	0.29935255	-0.1540995	4.14787727	5.39E-05	0.00295611	1.51310718	4.26828239	2.52927938
**SLC25A25**	-0.2029176	0.03413888	-4.1442167	5.47E-05	0.00298071	1.49960931	4.26200894	2.52567998
**COX7A2L**	-0.232831	-0.0078392	-4.1415074	5.53E-05	0.00299581	1.48962553	4.25736844	2.52348621
**FLNC**	0.4398063	-0.1205748	4.13932253	5.58E-05	0.00300484	1.48157805	4.25362775	2.52217855
**SLC25A15**	-0.2237748	0.02228987	-4.131938	5.74E-05	0.00307634	1.45440395	4.24099519	2.51196546
**TTC4**	0.15775691	-0.0033156	4.12318951	5.94E-05	0.00316646	1.42226132	4.22605033	2.49942667

**RPH3AL**	0.87722952	-0.0464743	4.17422193	6.01E-05	0.00317372	1.61303904	4.22110244	2.49843098
**PIAS2**	-0.3515126	-0.0265293	-4.1198093	6.02E-05	0.00317372	1.40985696	4.22028212	2.49843098
**NDUFS5**	-0.2453457	0.03691885	-4.1176587	6.07E-05	0.00318325	1.40196908	4.21661392	2.49712951
**TFDP2**	-0.3960483	0.00882824	-4.1222548	6.12E-05	0.0031892	1.45071087	4.21344827	2.49631776
**PDF**	-0.3001593	-0.0199381	-4.1118039	6.21E-05	0.00322222	1.38051217	4.2066347	2.49184541
**PMM1**	0.46440544	-0.0896215	4.10916978	6.28E-05	0.00323827	1.37086637	4.2021482	2.48968758
**MRFAP1**	-0.2096778	-0.0629503	-4.1019099	6.46E-05	0.00331398	1.34430798	4.18979393	2.47964955
**IFI44**	0.65505603	-0.4739013	4.09792866	6.73E-05	0.00339252	1.36248401	4.17224697	2.46947751
**ODR4**	-0.1782424	0.07919814	-4.0913545	6.73E-05	0.00339252	1.30576126	4.17185954	2.46947751
**SAMM50**	-0.1776895	0.07287665	-4.0911383	6.74E-05	0.00339252	1.30497264	4.17149258	2.46947751
**EDC4**	0.11264805	0.04657553	4.09054084	6.75E-05	0.00339252	1.30279336	4.17047852	2.46947751
**ZSWIM1**	-0.3298768	0.06885008	-4.125161	6.90E-05	0.00344714	1.48593347	4.16088988	2.46254144
**PDP1**	-0.1753109	0.06659058	-4.0837985	6.93E-05	0.00344714	1.27821759	4.15904194	2.46254144
**ATAD1**	-0.187465	0.01011304	-4.0809132	7.01E-05	0.00345446	1.26771059	4.1541519	2.46161963
**RFXANK**	-0.1586012	0.02558219	-4.0806263	7.02E-05	0.00345446	1.26666617	4.15366579	2.46161963
**ALKBH1**	-0.2545495	-0.0550254	-4.0622847	7.54E-05	0.00369144	1.20001939	4.12264036	2.43280436
**PYGO2**	0.32138889	0.14678996	4.06991489	7.68E-05	0.00370362	1.2474914	4.11464558	2.43137334
**HOOK1**	0.59043618	-0.005449	4.06553781	7.63E-05	0.00370362	1.24565493	4.11765544	2.43137334
**MRPL4**	-0.1640682	0.01712615	-4.0584715	7.65E-05	0.00370362	1.1861943	4.11620292	2.43137334
**TAGLN**	0.71452103	-0.465963	4.0540334	7.79E-05	0.00373586	1.17011653	4.10871587	2.42760969
**PYHIN1**	-0.5454294	-0.1228766	-4.0464176	8.02E-05	0.00382885	1.14256071	4.09588201	2.41693115
**TMEM9B**	-0.2705931	0.03268553	-4.0421809	8.15E-05	0.00387308	1.12724959	4.08875005	2.41194386
**HBZ**	0.97181796	-0.644456	4.05211078	8.25E-05	0.00390047	1.23981065	4.08355563	2.40888357
**PIAS4**	-0.1376393	0.02123973	-4.034371	8.40E-05	0.00395304	1.09905908	4.07561707	2.4030687
**CHDH**	-0.7657686	-0.1768235	-4.1140477	8.67E-05	0.00404123	1.23410266	4.06181796	2.39348608
**NIPSP2**	-0.185764	0.03768489	-4.0269036	8.65E-05	0.00404123	1.07214637	4.0630772	2.39348608
**ETFA**	-0.1833199	0.08331864	-4.0234364	8.76E-05	0.00404694	1.05966428	4.05726049	2.39287303
**TSFM**	-0.1546488	0.02811164	-4.0232983	8.77E-05	0.00404694	1.0591675	4.05702898	2.39287303
**HARS2**	-0.177148	0.05107418	-4.0150708	9.05E-05	0.00414902	1.02958428	4.04324119	2.38205397
**ATP5IF1**	-0.2035149	0.03605964	-4.0143737	9.08E-05	0.00414902	1.02707966	4.04207375	2.38205397
**OAS3**	0.38122528	-0.0692535	4.00787381	9.31E-05	0.00423418	1.00374665	4.03119697	2.3732306
**EEF1A2**	0.38421198	0.10974189	4.00620976	9.37E-05	0.00424139	0.99777804	4.02841441	2.37249178
**MAP4K3**	-0.2574631	-0.0491998	-4.0059043	9.59E-05	0.00430434	1.03251117	4.01795725	2.36609348
**ACOT13**	-0.2492162	0.03196253	-4.0005768	9.57E-05	0.00430434	0.97758865	4.01900136	2.36609348
**SUCLG1**	-0.1888096	0.06789475	-3.9980044	9.67E-05	0.00431661	0.96837663	4.01470597	2.36485749
**GPAM**	-0.2061726	0.06348629	-3.9884363	0.00010029	0.00445755	0.93415457	3.99874659	2.35090409
**HMGCL**	-0.2127314	0.0780377	-3.9847458	0.00010172	0.00450036	0.92097269	3.9925983	2.34675256
**MTIF3**	-0.1828922	0.07096113	-3.9810631	0.00010317	0.00453845	0.9078287	3.98646715	2.3430924
**AIMP2**	0.15467895	0.03330156	3.98016464	0.00010352	0.00453845	0.90462345	3.98497195	2.3430924
**TUBAL3**	0.40139025	0.16101259	4.02214737	0.00010906	0.00475595	0.98459713	3.96235435	2.32276236
**ZNF414**	0.34796993	-0.0174919	3.97041213	0.00010987	0.00475595	0.90685927	3.95911988	2.32276236
**TYW5**	0.15188791	0.05980426	3.96369939	0.00011025	0.00475595	0.84598944	3.95761445	2.32276236
**UQCRC2**	-0.1412453	0.04043063	-3.9623253	0.00011083	0.00475595	0.84110533	3.95533514	2.32276236
**SOD2**	-0.2562119	0.07278219	-3.9620522	0.00011095	0.00475595	0.84013452	3.95488207	2.32276236
**TRAPPC1**	0.12148637	0.0668164	3.95518491	0.00011389	0.00486065	0.81574691	3.94349968	2.31330589
**CRTC1**	0.30741868	-0.0291758	3.95116393	0.00011565	0.004914	0.80148337	3.93684162	2.30856524
**PSMB3**	0.1233721	0.0811426	3.94797604	0.00011707	0.00495223	0.7901835	3.9315665	2.30519912
**COCH**	0.77541081	-0.2649041	3.95430723	0.00012263	0.00512002	0.8815028	3.91141839	2.29072814
**RPAIN**	-0.3216982	-0.1070525	-3.9486473	0.00012227	0.00512002	0.81714782	3.91267176	2.29072814
**MTX2**	-0.157398	0.03299202	-3.9379247	0.00012163	0.00512002	0.75460432	3.91495444	2.29072814
**IBA57**	-0.2001713	0.10476038	-3.9281485	0.00012623	0.00524793	0.72007056	3.89882662	2.28001238
**SMAD1**	-0.4722142	-0.1536471	-3.9242671	0.00012811	0.00530292	0.70637933	3.89243154	2.27548522
**MRPL51**	-0.1860964	-0.001423	-3.9186309	0.00013087	0.00539427	0.6865182	3.88315345	2.26806707
**IFIT3**	0.47137471	-0.4389143	3.9118968	0.00013425	0.00549239	0.66281917	3.8720808	2.26023872
**TMED2**	-0.169302	0.11688197	-3.9105145	0.00013496	0.00549239	0.65795864	3.86980965	2.26023872
**CCDC77**	-0.1552147	0.01131581	-3.9102943	0.00013507	0.00549239	0.65718432	3.86944783	2.26023872
**CNOT6**	0.15773638	0.02462307	3.90939093	0.00013553	0.00549239	0.65400887	3.867964	2.26023872
**HELZ2**	0.35128929	-0.0592979	3.90805097	0.00013622	0.00549719	0.64929967	3.86576341	2.25985908
**ZKSCAN3**	-0.3984238	0.08667932	-3.9202413	0.00013928	0.00557428	0.82823931	3.85609623	2.25381104
**DECR1**	-0.1747374	-0.0152041	-3.903095	0.00013879	0.00557428	0.63189377	3.8576291	2.25381104
**FBXO11**	-0.1292699	0.00895805	-3.8982927	0.00014133	0.00563291	0.61504497	3.84975418	2.24926731
**GFI1B**	0.38758713	0.09865857	3.90069514	0.00014301	0.00567617	0.66268296	3.8446407	2.24594474
**FBXO42**	-0.1523874	0.05228181	-3.8933095	0.00014402	0.00567767	0.59757973	3.84159015	2.24583001
**NDEL1**	0.12815836	0.01799388	3.89292843	0.00014422	0.00567767	0.59624491	3.84096616	2.24583001
**F13A1**	-0.5156913	-0.1849293	-3.8916021	0.00014495	0.00568293	0.59159973	3.8387946	2.24542748

**CHST2**	0.52115125	-0.0300998	3.89143853	0.00014806	0.00575879	0.63052743	3.82955049	2.23966883
**ACAD10**	-0.1686959	0.04545583	-3.8859332	0.00014807	0.00575879	0.57176082	3.82951938	2.23966883
**GMCL1**	-0.2807241	0.00368289	-3.9007184	0.00014933	0.00577148	0.69200341	3.8258553	2.238713
**COG5**	0.09827204	0.0269667	3.88321485	0.0001496	0.00577148	0.56225614	3.82507522	2.238713
**RPUSD4**	-0.2130591	-0.0089021	-3.8808733	0.00015092	0.00579935	0.55407337	3.82124892	2.23662041
**NDUFA8**	-0.1487246	0.03098896	-3.8771693	0.00015304	0.00585736	0.5411377	3.81519966	2.23229797
**RHOT2**	-0.161847	0.08851248	-3.8760181	0.0001537	0.00585951	0.53711917	3.81332032	2.23213861
**HGF**	0.47967236	-0.4249567	3.87557304	0.00015713	0.00594313	0.5755591	3.80374646	2.22598441
**MRPS11**	-0.1932559	-0.0094461	-3.8708928	0.00015669	0.00594313	0.51924124	3.80495868	2.22598441
**GAS6**	-0.9377608	-0.0369461	-3.9000938	0.0001598	0.00602072	0.75385022	3.79641406	2.2203518
**SP140L**	-0.1776162	-0.0136969	-3.862599	0.00016164	0.00605295	0.49035208	3.79144471	2.21803312
**MPC2**	-0.2014238	-0.0049551	-3.8621512	0.00016191	0.00605295	0.48879369	3.79071564	2.21803312
**MXD3**	-0.4763694	-0.0616832	-3.9390214	0.00016375	0.00606198	0.67936396	3.78581168	2.21738562
**HIBADH**	-0.2288246	0.03636202	-3.8589929	0.00016384	0.00606198	0.47780707	3.78557544	2.21738562
**KRT8**	-0.5541268	-0.1080485	-3.8578857	0.00016452	0.00606198	0.47395725	3.78377416	2.21738562
**OASL**	0.46070352	-0.2554269	3.85764738	0.00016467	0.00606198	0.47312887	3.78338656	2.21738562
**RNF25**	-0.1441477	-0.0258881	-3.8537236	0.00016711	0.0061283	0.45949385	3.77700648	2.21266
**HACD2**	-0.2198245	0.03452262	-3.8521822	0.00016807	0.0061404	0.4541406	3.77450141	2.21180311
**GOSR2**	-0.7380365	-0.0357306	-3.9282548	0.00017386	0.00628038	0.69747878	3.75980528	2.20201431
**GTF3C6**	-0.1443205	-0.0026485	-3.8441134	0.00017322	0.00628038	0.42614756	3.7614004	2.20201431
**GTPBP8**	-0.2039482	0.03720706	-3.8434466	0.00017365	0.00628038	0.42383616	3.76031852	2.20201431
**ARID5A**	-0.5358408	0.07671702	-3.8671687	0.00017506	0.00630029	0.63565585	3.75680673	2.20063929
**RASGRF2**	-0.5052403	-0.1340028	-3.909628	0.00018041	0.00646871	0.58826225	3.74373241	2.18918246
**CLIP4**	0.18824889	0.08809214	3.84767528	0.00018223	0.00650984	0.4145356	3.73936848	2.18643002
**POC1B**	-0.1721986	0.05908848	-3.8284274	0.00018366	0.00653402	0.37186682	3.73598867	2.18481982
**MAPKAPK5**	0.30730985	0.09959065	3.82753984	0.00018427	0.00653402	0.36880114	3.73455314	2.18481982
**VANGL1**	-0.3675527	0.02658254	-3.8349033	0.00018641	0.00658565	0.48106588	3.72954068	2.18140109
**CNN3**	0.67042264	0.13684885	3.83321671	0.00018757	0.00660039	0.47534478	3.7268359	2.18043064
**FAU**	0.20862493	0.03003658	3.82187745	0.00018819	0.00660039	0.34925585	3.72540018	2.18043064
**SLC7A6**	-0.7690463	0.02504289	-3.8602289	0.00019719	0.00689085	0.53977235	3.70512056	2.16172741
**KHDRBS3**	-0.6604073	-0.1356547	-3.8054151	0.00020006	0.00696589	0.2925683	3.69884595	2.15702348
**PIP5K1A**	-0.1558872	-0.0327056	-3.8003628	0.00020384	0.00707203	0.27521178	3.69071328	2.15045585
**FAF2**	-0.0908979	0.01507824	-3.7991386	0.00020477	0.00707871	0.27100924	3.68874396	2.15004593
**POU2F2**	0.69084756	-0.2174444	3.80818596	0.0002062	0.00710291	0.39715999	3.68570784	2.14856364
**GLRX2**	-0.2926218	-0.0536483	-3.8229298	0.00021	0.00711876	0.3967822	3.67777671	2.14759557
**PRUNE2**	0.6185202	-0.0591489	3.80449938	0.00020901	0.00711876	0.38474795	3.67982976	2.14759557
**C5orf51**	-0.166021	0.01508147	-3.7929812	0.00020949	0.00711876	0.24988797	3.67884546	2.14759557
**EIF3G**	0.10948252	-0.0217433	3.79226448	0.00021004	0.00711876	0.24743114	3.67769396	2.14759557
**IMPDH2**	0.13323544	-0.0269937	3.79186542	0.00021035	0.00711876	0.24606349	3.67705294	2.14759557
**LRCH4**	0.11515006	0.00600068	3.78635829	0.00021468	0.00723164	0.22720158	3.66821164	2.14076298
**CNBP**	0.34748411	0.13003045	3.78571738	0.00021519	0.00723164	0.22500795	3.66718332	2.14076298
**PEX14**	-0.138662	-0.0211302	-3.7806039	0.00021929	0.00734389	0.20751722	3.65898344	2.13407369
**DEF8**	0.33134292	0.0324449	3.784605	0.00022481	0.0074004	0.31793594	3.64817932	2.13074471
**PAWR**	-0.7350384	-0.3177536	-3.7823048	0.00022203	0.0074004	0.25614416	3.65359377	2.13074471
**FAM173A**	-0.3797392	-0.0782928	-3.7800562	0.00022387	0.0074004	0.24852257	3.65000647	2.13074471
**SLC35F6**	-0.3080054	-0.0569862	-3.7760846	0.00022298	0.0074004	0.19207529	3.65174311	2.13074471
**TARBP2**	0.15865434	0.05872206	3.77389276	0.00022478	0.0074004	0.18459174	3.64823395	2.13074471
**ANGEL2**	-0.1327839	0.06244698	-3.7678754	0.00022982	0.00753958	0.1640652	3.63860763	2.12265273
**PIP5K1C**	-0.2577974	0.03620165	-3.7636494	0.00023342	0.00763179	0.14966562	3.63185373	2.11737351
**MRPL53**	-0.1933871	-0.027272	-3.7620086	0.00023484	0.00765205	0.14407857	3.62923299	2.11622247
**DTNB**	-0.2725871	-0.0270341	-3.762774	0.00023851	0.00774567	0.19006909	3.62248653	2.11094075
**TMEM70**	-0.283121	0.04270439	-3.7565689	0.00023958	0.00775417	0.12557019	3.62055037	2.1104644
**FAM210B**	-0.3982113	0.08395784	-3.7566591	0.00024391	0.00784169	0.16943963	3.61277109	2.10559011
**DJC30**	-0.2716975	-0.0402424	-3.7517438	0.00024386	0.00784169	0.10917157	3.6128564	2.10559011
**LRRC8B**	-0.3407313	-0.0376873	-3.7630494	0.00024821	0.00793648	0.2263012	3.60518132	2.10037202
**TIMM22**	-0.29474	-0.0095549	-3.7466031	0.0002485	0.00793648	0.09172008	3.6046673	2.10037202
**C5orf63**	-0.8746974	0.1769099	-3.778438	0.00025483	0.0081117	0.29962894	3.59374749	2.09088789
**ZBED4**	-0.2146595	-0.0406935	-3.7388394	0.00025567	0.00811173	0.06540173	3.59231523	2.09088658
**TUBB4B**	0.2218864	0.09304868	3.73624091	0.00025812	0.00815835	0.05660345	3.58818531	2.08839757
**AUH**	-0.2476574	0.05228741	-3.7354813	0.00025883	0.00815835	0.05403234	3.58697838	2.08839757
**ZNF480**	0.58709162	0.03779798	3.81070991	0.00026003	0.00816949	0.34663057	3.58496899	2.08780514
**YARS2**	-0.1748016	0.03523735	-3.7324733	0.0002617	0.00819202	0.04385573	3.58220099	2.08660905
**SNRPF**	-0.130858	-0.0088803	-3.7316872	0.00026245	0.00819202	0.04119703	3.58095281	2.08660905
**KIAA2013**	-0.145889	0.03936958	-3.7279091	0.0002661	0.0082791	0.02842682	3.57495716	2.08201662
**MT-ND2**	-0.259933	0.0572985	-3.722907	0.000271	0.00840458	0.01153592	3.56702587	2.07548402

**KIF3C**	0.47906824	-0.002225	3.74338723	0.00027332	0.00844927	0.28268938	3.56332859	2.07318095
**BTBD1**	0.21412827	0.0601179	3.73249459	0.00027646	0.00851889	0.12659723	3.55837481	2.06961692
**BTG3**	-0.49979	-0.0452088	-3.7628158	0.00027855	0.00855611	0.16812063	3.55509638	2.0677238
**PTGS2**	-0.5355378	-0.0032901	-3.7385482	0.00028523	0.00873359	0.06865087	3.54479913	2.05880745
**DESI1**	0.46238092	-0.0975763	3.75352316	0.00028653	0.00874551	0.20052575	3.54283019	2.05821504
**NLRP12**	0.7377399	-0.2692118	3.72383315	0.00029228	0.00879933	0.16179915	3.5342027	2.0555503
**ZUFSP**	0.29392506	-0.0048497	3.71671437	0.00029412	0.00879933	0.16100787	3.53148012	2.0555503
**ZCCHC2**	0.31859165	-0.0237568	3.71546143	0.00029468	0.00879933	0.149027	3.53065001	2.0555503
**PPP1R15A**	0.47431295	0.00584185	3.73278413	0.00029114	0.00879933	0.10581853	3.53589559	2.0555503
**KRT80**	0.42517804	0.19303981	3.75092408	0.00029307	0.00879933	0.07890089	3.53302992	2.0555503
**MTX1**	-0.1209165	0.0228411	-3.7042778	0.00029003	0.00879933	-0.0512023	3.53755662	2.0555503
**GZF1**	0.22353642	0.06706996	3.70389929	0.00029043	0.00879933	-0.0524744	3.53695897	2.0555503
**GLB1L2**	-0.6093764	-0.1065033	-3.7446609	0.00029688	0.0088378	0.16950529	3.52741313	2.05365591
**LAMA4**	0.5496157	-0.1618848	3.69963171	0.00030011	0.00886448	-0.0216087	3.52271858	2.05234666
**GON4L**	0.23026884	0.05777713	3.69866289	0.00030116	0.00886448	-0.0248334	3.52119736	2.05234666
**TBC1D24**	0.16692914	0.03223469	3.69496461	0.00030001	0.00886448	-0.0824676	3.52286494	2.05234666
**TAMM41**	-0.2041158	0.02534623	-3.6936384	0.00030146	0.00886448	-0.0869144	3.52077504	2.05234666
**PNPT1**	-0.167213	0.03464389	-3.6926379	0.00030255	0.00886967	-0.0902681	3.51919884	2.0520925
**CCNK**	0.0824979	0.05024481	3.68394898	0.00031223	0.0091119	-0.1193625	3.50552278	2.04039099
**CKAP2L**	0.37965803	-0.1632982	3.68353083	0.0003127	0.0091119	-0.1207612	3.50486523	2.04039099
**RANBP1**	0.13793187	-0.0611375	3.67901724	0.00031785	0.00923407	-0.1358504	3.49777097	2.03460682
**ACSF3**	-0.168361	0.04288951	-3.6751654	0.00032231	0.00933547	-0.148715	3.49172193	2.02986393
**LETMD1**	-0.1984509	0.04451036	-3.6733729	0.00032441	0.00936801	-0.1546979	3.48890847	2.02835271
**RPL38**	0.13445168	0.03949767	3.67165626	0.00032643	0.00939815	-0.1604252	3.48621507	2.02695765
**OTUD5**	0.24413139	0.09620944	3.67953755	0.00032885	0.00943158	-0.0301634	3.48300003	2.02541561
**FASTKD5**	-0.2010089	0.04341519	-3.6684314	0.00033025	0.00943158	-0.1711783	3.48115778	2.02541561
**EIF2S3**	0.16856756	0.07995626	3.66820391	0.00033052	0.00943158	-0.1719366	3.48080114	2.02541561
**POMGNT1**	-0.169316	0.03140944	-3.6669444	0.00033203	0.0094466	-0.1761339	3.47882699	2.02472446
**GBA2**	-0.1853801	0.03000126	-3.6643116	0.0003352	0.0094811	-0.184904	3.47470181	2.02314106
**CNPY4**	-0.1966714	0.05683392	-3.6643034	0.00033521	0.0094811	-0.1849314	3.47468891	2.02314106
**SLC25A23**	-0.5045095	0.04596604	-3.6912458	0.00033728	0.00951185	0.03928923	3.47201096	2.02173484
**NDUFV2**	-0.1434645	0.05383469	-3.6601409	0.00034027	0.00956834	-0.1987859	3.46817152	2.01916341
**POGK**	-0.1569914	0.00584747	-3.6586118	0.00034215	0.00959324	-0.2038724	3.46577858	2.0180348
**JAGN1**	-0.1391495	0.08840834	-3.656462	0.00034481	0.00963979	-0.2110202	3.46241569	2.01593255
**NDUFB6**	-0.1134397	0.04992732	-3.6537158	0.00034824	0.00968038	-0.2201456	3.45812204	2.01410761
**SLC25A4**	-0.3882133	-0.0852981	-3.6536892	0.00034827	0.00968038	-0.2202341	3.45808037	2.01410761
**LGALSL**	0.41208237	-0.1642021	3.64766387	0.0003559	0.00984943	-0.2402357	3.44866809	2.00658888
**GMNN**	0.38987328	-0.2319882	3.64727799	0.0003564	0.00984943	-0.2415158	3.44806569	2.00658888
**TUBB2A**	0.47855521	0.12085	3.65062684	0.00035798	0.00986068	-0.1838361	3.44613593	2.00609332
**CARM1**	0.1083318	0.03737684	3.64537041	0.00035885	0.00986068	-0.2478417	3.44508844	2.00609332
**CLIC3**	-0.2664814	0.04423974	-3.6428886	0.00036206	0.00992071	-0.256068	3.44121663	2.00345706
**IL13RA1**	-0.5912912	-0.1172174	-3.6779881	0.00036444	0.00992952	-0.022691	3.4383705	2.00307153
**SLC25A36**	-0.2244515	0.03057896	-3.6416758	0.00036364	0.00992952	-0.260086	3.43932535	2.00307153
**IVD**	-0.1327829	0.03212739	-3.6395408	0.00036644	0.00995582	-0.2671567	3.43599703	2.00192315
**ANKZF1**	0.10212951	0.04926857	3.63337154	0.00037464	0.01014997	-0.2875688	3.42638751	1.99353528
**MAIP1**	-0.1947485	0.03782117	-3.6283272	0.00038147	0.01030611	-0.304237	3.41853921	1.98690519
**CYC1**	-0.1222337	0.01760908	-3.6249305	0.00038614	0.01040304	-0.3154498	3.4132589	1.9828397
**SNX16**	-0.2093607	-0.0329223	-3.6234149	0.00038824	0.01043046	-0.3204499	3.41090412	1.98169633
**ZXDC**	0.30279081	0.1148577	3.62211063	0.00039636	0.01061903	-0.2774045	3.40191488	1.97391515
**TMSB10**	0.45546459	-0.2828081	3.61915284	0.00040055	0.01065511	-0.2870745	3.39734262	1.97244217
**GSK3A**	-0.2208779	-0.0564763	-3.6148172	0.00040035	0.01065511	-0.348782	3.39755905	1.97244217
**MTMR9**	-0.1544308	-0.0380341	-3.6143314	0.00040105	0.01065511	-0.3503811	3.39680573	1.97244217
**SSSCA1**	0.21541299	0.08248312	3.61358151	0.00040212	0.01065511	-0.3528492	3.39564302	1.97244217
**UQCRC1**	-0.1208552	0.0501089	-3.604941	0.00041471	0.01095849	-0.3812565	3.38225849	1.96024912
**PRR15**	0.62317511	-0.3555891	3.62383165	0.00041649	0.01097555	-0.2987956	3.38039488	1.95957373
**SLAMF6**	0.28253215	0.10222344	3.60602983	0.00041967	0.01102927	-0.329898	3.37708947	1.95745331
**IMMT**	-0.0983941	0.03476664	-3.6002845	0.00042164	0.01105094	-0.3965415	3.37505529	1.95660088
**C1orf122**	0.19385766	0.0431807	3.60063327	0.00042778	0.0111604	-0.3474703	3.36877639	1.95232033
**SUOX**	-0.3736769	0.01026066	-3.5946073	0.00043025	0.0111604	-0.4151544	3.36628235	1.95232033
**TMEM106B**	-0.1638505	0.05737294	-3.5945273	0.00043037	0.0111604	-0.4154164	3.36615886	1.95232033
**FIS1**	-0.1847279	0.01789028	-3.594476	0.00043045	0.0111604	-0.4155845	3.36607962	1.95232033
**ZFAND3**	-0.2051466	-0.0783811	-3.5932792	0.00043228	0.01117794	-0.4195049	3.3642316	1.9516382
**TTLL12**	0.10031482	-0.0125131	3.59237575	0.00043367	0.01118392	-0.4224638	3.36283675	1.95140612
**KTI12**	0.10770616	0.00428949	3.59008358	0.00043722	0.01123765	-0.4299677	3.35929921	1.94932433
**PPM1L**	-0.233221	-0.0009844	-3.5895259	0.00043809	0.01123765	-0.4317929	3.35843871	1.94932433

**BOLA3**	-0.2502416	0.05227273	-3.5864127	0.00044296	0.01133115	-0.4419766	3.35363737	1.94572609
**TUBA4B**	0.17935365	0.06352881	3.58569866	0.00044408	0.01133115	-0.4443114	3.35253653	1.94572609
**RAB2B**	-0.1675143	0.02833921	-3.5829064	0.0004485	0.01141378	-0.4534374	3.34823336	1.94257034
**ATRAID**	-0.4395147	-0.0362901	-3.6258233	0.00045014	0.01141709	-0.1398771	3.34665659	1.94244471
**C22orf39**	-0.586891	-0.0145245	-3.6025857	0.000451	0.01141709	-0.2180667	3.34582196	1.94244471
**ZNF511**	-0.1820894	-0.0239543	-3.5785726	0.00045545	0.01149951	-0.4675898	3.34155935	1.93932048
**CPD**	-0.272128	-0.0256203	-3.5720657	0.00046607	0.0117369	-0.4888113	3.33155	1.93044654
**MTUS2**	-0.3576719	-0.0869313	-3.5867856	0.00047287	0.01187474	-0.4540705	3.32525823	1.92537573
**FBXW9**	0.20861978	0.00597574	3.56729326	0.000474	0.01187474	-0.5043552	3.32421723	1.92537573
**SUMO2**	0.13707506	-0.0423388	3.56413434	0.00047933	0.01197448	-0.5146341	3.31936763	1.92174321
**NIPSP1**	-0.2237786	0.07282095	-3.5634611	0.00048047	0.01197448	-0.5168237	3.31833447	1.92174321
**TP53**	0.65023405	-0.4053695	3.56600754	0.00048344	0.01201756	-0.4596921	3.31565407	1.92018356
**SLC25A16**	-0.2639222	-0.0308209	-3.5577125	0.00049032	0.01215721	-0.5355063	3.30951855	1.91516592
**RUBCN**	-0.1226851	0.01435675	-3.5563808	0.00049263	0.01218316	-0.5398307	3.30747772	1.9142401
**TMEM147**	-0.171418	0.01366093	-3.5484973	0.00050651	0.01249445	-0.5654014	3.29540828	1.90328281
**HSD17B10**	-0.1535468	0.10047725	-3.5463226	0.00051041	0.01253372	-0.5724468	3.29208231	1.90191998
**ISG20**	0.35675385	-0.204973	3.54615736	0.00051071	0.01253372	-0.5729819	3.29182966	1.90191998
**GPR108**	-0.2083799	0.05374129	-3.5444472	0.00051379	0.01257739	-0.5785194	3.28921535	1.90040934
**TMEM223**	-0.1936784	-0.0512153	-3.5452247	0.00052006	0.01266863	-0.5266099	3.28394978	1.89727041
**L2HGDH**	-0.1985109	0.03999417	-3.5409535	0.00052014	0.01266863	-0.5898249	3.28387745	1.89727041
**CRYAB**	0.78462128	0.01594502	3.60558061	0.00052216	0.01268395	-0.2577713	3.282193	1.89674538
**ZNF121**	-0.3913308	-0.028917	-3.5507491	0.00052735	0.01268395	-0.4539955	3.27790324	1.89674538
**LM**	-0.3593345	-0.1517521	-3.5388368	0.00052403	0.01268395	-0.5966702	3.28064516	1.89674538
**RPL7A**	0.10062384	0.02845322	3.53763427	0.00052625	0.01268395	-0.6005573	3.27880961	1.89674538
**NDUFS1**	-0.1221854	0.06043824	-3.5376266	0.00052626	0.01268395	-0.6005819	3.27879797	1.89674538
**STXBP4**	-0.4555175	-0.0336633	-3.5544001	0.00053179	0.01271324	-0.2952505	3.27426174	1.89574376
**CD276**	-0.442852	-0.0744123	-3.596711	0.00053214	0.01271324	-0.3363507	3.27397486	1.89574376
**MRPS16**	-0.1441094	-0.0208119	-3.5342621	0.00053252	0.01271324	-0.6114518	3.27366463	1.89574376
**MPDU1**	-0.2423524	0.08226239	-3.5314362	0.00053783	0.01279529	-0.6205749	3.26935582	1.89294991
**TMC6**	0.27624728	0.03425709	3.53044345	0.00053971	0.01279529	-0.6237782	3.26784279	1.89294991
**POLR2M**	-0.2674683	-0.1049965	-3.530322	0.00053994	0.01279529	-0.62417	3.26765774	1.89294991
**TCF19**	0.4283597	-0.0193253	3.5697689	0.00054496	0.01285529	-0.305424	3.26363179	1.89091825
**VPS13D**	-0.0839947	0.04237468	-3.5275911	0.00054513	0.01285529	-0.632978	3.26349718	1.89091825
**SLC29A3**	-0.2980153	-0.0459999	-3.5251267	0.00054986	0.01293522	-0.6409214	3.25974472	1.88822635
**SUSD1**	-0.273254	0.04695464	-3.5224196	0.0005551	0.01302673	-0.6496414	3.25562505	1.88516464
**TSC22D2**	0.13518771	0.07380967	3.5212805	0.00055732	0.01304707	-0.6533091	3.25389217	1.88448716
**COX5A**	-0.1328541	0.00460869	-3.5201857	0.00055946	0.01306017	-0.656833	3.25222716	1.88405131
**ACOT9**	-0.1495489	0.0384958	-3.519611	0.00056059	0.01306017	-0.6586827	3.2513532	1.88405131
**COQ9**	-0.1771271	0.0369696	-3.5146853	0.00057034	0.01325521	-0.6745237	3.24386765	1.87761351
**SLC25A10**	-0.4318812	0.01255078	-3.5138319	0.00057204	0.01326287	-0.6772666	3.24257141	1.87736251
**ATXN7L2**	-0.265143	0.03496675	-3.5152004	0.00057758	0.01329539	-0.6227	3.23838728	1.87629907
**TACO1**	-0.1312816	0.04636764	-3.5123686	0.00057498	0.01329539	-0.681968	3.2403495	1.87629907
**NDUFA2**	-0.1974044	0.04829634	-3.5111095	0.00057751	0.01329539	-0.686012	3.23843817	1.87629907
**VDAC1**	-0.1479699	0.06538273	-3.5100598	0.00057964	0.01331091	-0.6893825	3.23684514	1.8757922
**MLXIP**	-0.1413281	-0.0316033	-3.5087735	0.00058225	0.0133391	-0.6935115	3.23489353	1.8748734
**LIAS**	-0.134762	0.02182633	-3.5048153	0.00059035	0.01349269	-0.706209	3.22889136	1.86990158
**PCED1B**	-0.6411492	0.09576709	-3.5675747	0.0005926	0.01349671	-0.3671524	3.22723937	1.86977215
**DPYSL5**	-0.1858322	0.05657435	-3.5219355	0.00059332	0.01349671	-0.6607612	3.22670853	1.86977215
**PKIB**	1.29486226	-0.5609294	3.53322005	0.00059996	0.01361567	-0.4615937	3.22187416	1.86596086
**CTNND1**	-0.1783568	-0.0241807	-3.4991619	0.00060211	0.01363218	-0.7243236	3.22032719	1.86543456
**ZNF160**	-0.3376458	0.00868315	-3.5227144	0.00060385	0.01363974	-0.6213627	3.21906828	1.86519392
**RMI2**	0.14987654	-0.0190788	3.49763434	0.00060532	0.01364092	-0.7292138	3.21801493	1.86515647
**CDK4**	0.23138001	0.00896075	3.49679256	0.0006071	0.0136491	-0.7319079	3.21674103	1.86489609
**METTL26**	0.15330445	0.0281102	3.49100837	0.00061945	0.01389441	-0.7504047	3.20799377	1.85715999
**NUP54**	-0.0861381	0.0516186	-3.4903082	0.00062096	0.01389598	-0.7526418	3.20693572	1.85711076
**SNX7**	0.54906179	0.07333742	3.53967449	0.00062565	0.01396852	-0.5581632	3.20366798	1.85484949
**EPHX2**	-0.4541487	0.10889484	-3.4822562	0.00063859	0.01420564	-0.7783429	3.19477845	1.84753906
**IP6K1**	-0.1208777	0.03318231	-3.4819732	0.00063922	0.01420564	-0.7792452	3.19435156	1.84753906
**TMEM69**	-0.4974572	-0.0754762	-3.5044063	0.00064278	0.014252	-0.6253931	3.19193731	1.84612433
**IARS**	0.09078452	0.01130731	3.47936404	0.00064503	0.01426917	-0.7875616	3.19041694	1.8456012
**LLGL1**	-0.1198186	0.03453155	-3.4708263	0.00066442	0.01466438	-0.8147377	3.17755709	1.8337363
**SOGA1**	-0.1690781	-0.0614332	-3.4677128	0.00067162	0.01472764	-0.8246337	3.17287333	1.83186681
**TMEM39B**	-0.1672686	0.00242543	-3.4676791	0.0006717	0.01472764	-0.8247409	3.17282261	1.83186681
**DAP3**	-0.1372812	0.02308002	-3.4676085	0.00067187	0.01472764	-0.8249653	3.17271637	1.83186681
**CHD2**	-0.1247546	0.02928836	-3.4649696	0.00067803	0.01480909	-0.8333466	3.16874914	1.82947155

**PTPMT1**	-0.2142105	0.04656447	-3.4642664	0.00067969	0.01480909	-0.8355792	3.16769226	1.82947155
**CHCHD6**	-0.2303567	-0.0117619	-3.464052	0.00068019	0.01480909	-0.8362599	3.16737006	1.82947155
**GTDC1**	0.30924668	0.12001799	3.4663284	0.00068418	0.01486236	-0.7776272	3.16483148	1.82791222
**ZNF440**	-0.4957951	0.01533497	-3.4877758	0.00069751	0.01501666	-0.6078062	3.15645079	1.82342658
**TMEM19**	-0.2022383	0.08666016	-3.4882523	0.00069639	0.01501666	-0.7697553	3.15714808	1.82342658
**GNPT1**	0.19163678	-0.0043452	3.45748891	0.0006958	0.01501666	-0.8570765	3.15751474	1.82342658
**RPS25**	0.13605332	0.0231817	3.45730157	0.00069625	0.01501666	-0.8576702	3.15723363	1.82342658
**RBM7**	0.25355537	-0.0732042	3.45992724	0.00069943	0.01502459	-0.7977826	3.15525328	1.82319739
**HIKESHI**	-0.1483748	0.07634721	-3.4493036	0.00071574	0.01530335	-0.8829909	3.14524287	1.81521343
**PRPF40B**	0.26207035	0.08291091	3.44911366	0.00071621	0.01530335	-0.8835916	3.14495836	1.81521343
**HSD17B7**	-0.1727836	0.05717876	-3.4487257	0.00071717	0.01530335	-0.8848186	3.14437722	1.81521343
**LYRM4**	-0.2124498	0.01505468	-3.4451823	0.00072599	0.01545724	-0.8960189	3.13907208	1.81086805
**PSMB2**	0.0987147	0.06454474	3.44237498	0.00073304	0.01552964	-0.9048853	3.13487197	1.80883852
**RLF**	-0.1378744	0.01288938	-3.441933	0.00073416	0.01552964	-0.9062806	3.13421094	1.80883852
**THEM6**	-0.1478196	0.04872322	-3.4419096	0.00073422	0.01552964	-0.9063545	3.13417591	1.80883852
**MAZ**	-0.2013768	-0.0003865	-3.4343427	0.00075358	0.01590441	-0.9302198	3.12286844	1.79848241
**NEK9**	0.09259031	0.04435586	3.43315462	0.00075667	0.01593463	-0.9339628	3.12109473	1.79765797
**MX1**	0.63005638	-0.8141458	3.43155137	0.00076085	0.01598776	-0.939012	3.11870191	1.79621236
**FBXO18**	-0.1328327	0.02888636	-3.4301263	0.00076458	0.01599894	-0.9434983	3.11657574	1.79590873
**GATB**	-0.1224988	0.00574778	-3.4300823	0.0007647	0.01599894	-0.9436369	3.11651004	1.79590873
**JAK1**	-0.112427	0.03348919	-3.4270545	0.00077269	0.01613115	-0.9531633	3.11199486	1.7923346
**DCAF4**	0.23868172	0.02538525	3.42967098	0.00078616	0.01637697	-0.833653	3.10448756	1.78576632
**OXLD1**	-0.1726404	0.04994948	-3.4211003	0.00078863	0.01639306	-0.971876	3.10312435	1.7853401
**PRSS21**	0.6601095	-0.2715621	3.42369901	0.00079197	0.01642706	-0.9112561	3.10128944	1.78444016
**GTPBP3**	-0.1524616	0.05065459	-3.4190073	0.00079431	0.01644021	-0.9784473	3.10000885	1.78409253
**CEBPZOS**	-0.3753709	-0.0805931	-3.4773443	0.00079755	0.01647187	-0.7606451	3.09824249	1.78325714
**SLC25A35**	-0.2814317	-0.0303247	-3.4152848	0.0008045	0.01658002	-0.9901259	3.0944714	1.78041502
**MT-CO2**	-0.149829	0.03528894	-3.4144	0.00080695	0.01659486	-0.9929002	3.09315585	1.78002646
**CLIC4**	-0.2494133	0.0025091	-3.4129855	0.00081086	0.01663991	-0.9973338	3.09105337	1.778849
**EPCAM**	-0.7986022	-0.1604728	-3.4696881	0.00082173	0.01665758	-0.7215878	3.08527344	1.77838799
**SLC19A1**	0.28671587	-0.007343	3.41779293	0.00081999	0.01665758	-0.8618959	3.08619043	1.77838799
**GGNBP2**	-0.1642727	-0.0706258	-3.4110592	0.00081622	0.01665758	-1.0033694	3.08819106	1.77838799
**DJC11**	-0.1034025	0.03521436	-3.4106997	0.00081723	0.01665758	-1.0044956	3.08765695	1.77838799
**JARID2**	-0.1768324	-0.0232847	-3.4101926	0.00081865	0.01665758	-1.0060838	3.08690375	1.77838799
**SLC25A13**	-0.2065954	-0.0013802	-3.4089668	0.00082209	0.01665758	-1.0099221	3.08508327	1.77838799
**STRADB**	0.3928072	0.11940729	3.42371518	0.00083068	0.01672642	-0.6807733	3.08056384	1.77659712
**CCSAP**	-0.2083351	-0.0292514	-3.4102469	0.00082792	0.01672642	-0.9596555	3.08201414	1.77659712
**TXNL4A**	-0.1053039	0.04256619	-3.4063375	0.00082951	0.01672642	-1.0181512	3.0811801	1.77659712
**CMTR2**	0.14883766	0.08500957	3.40510141	0.00083302	0.01673021	-1.0220181	3.07934589	1.77649873
**MRPL22**	-0.1335432	0.02374601	-3.4042388	0.00083548	0.01673021	-1.0247157	3.07806622	1.77649873
**HACD3**	-0.1297676	0.05469648	-3.4040283	0.00083608	0.01673021	-1.025374	3.07775393	1.77649873
**CDK7**	0.09616071	-0.0099177	3.40190657	0.00084216	0.01681697	-1.0320067	3.07460745	1.77425235
**SLC25A28**	-0.5476191	-0.1216184	-3.4299773	0.00084655	0.01686979	-0.6904294	3.07234726	1.77289039
**DJC28**	-0.6559144	0.00729948	-3.4356051	0.00085422	0.01693973	-0.7803007	3.06842965	1.77109352
**NDUFV1**	-0.1335186	0.05066914	-3.3985517	0.00085185	0.01693973	-1.0424868	3.06963526	1.77109352
**CLP1**	0.09693263	0.05221318	3.3973579	0.00085533	0.01693973	-1.046214	3.06786679	1.77109352
**CSNK1G3**	-0.2626362	0.03328575	-3.397996	0.00086446	0.01706336	-0.9911435	3.06325687	1.76793555
**FAM126A**	-0.1795518	-0.0269362	-3.3934694	0.00086674	0.01706336	-1.0583462	3.06210981	1.76793555
**ISOC2**	-0.1577395	0.05643101	-3.3934234	0.00086688	0.01706336	-1.0584897	3.0620417	1.76793555
**MDH2**	-0.1331748	0.03708972	-3.3926958	0.00086903	0.01707087	-1.0607586	3.060965	1.76774426
**FBXL15**	-0.2485646	-0.0282393	-3.4158034	0.00087116	0.01707772	-0.8908203	3.05990034	1.76757008
**MTRF1L**	-0.1512313	0.0862169	-3.3913491	0.00087303	0.01707772	-1.0649568	3.0589726	1.76757008
**ZBTB44**	-0.1808768	-0.0037916	-3.3907898	0.00087469	0.01707772	-1.0666998	3.05814537	1.76757008
**HSDL2**	-0.1578503	0.11513845	-3.3891056	0.00087972	0.01714124	-1.0719472	3.05565489	1.76595786
**LAPTM4A**	0.30039105	-0.0750096	3.39176976	0.00088293	0.01716899	-1.0104176	3.05407573	1.76525518
**FAM122A**	0.1630418	-0.0166632	3.38935393	0.00089019	0.01724153	-1.017888	3.0505167	1.76342424
**ZFAT**	-0.1157313	0.04810814	-3.3854967	0.00089059	0.01724153	-1.0831841	3.05032117	1.76342424
**ACAD8**	-0.2050439	0.10901673	-3.385026	0.00089202	0.01724153	-1.0846487	3.04962593	1.76342424
**VDAC2**	-0.1519382	0.06217118	-3.3820449	0.00090111	0.01738235	-1.0939218	3.04522379	1.75989156
**CCDC90B**	-0.1483434	0.06156169	-3.3813608	0.0009032	0.01738804	-1.0960488	3.04421398	1.75974948
**COQ8B**	-0.1741893	0.04278491	-3.3791146	0.00091012	0.01748635	-1.10303	3.04089944	1.75730093
**DYNLRB1**	0.11852459	0.02417802	3.37707711	0.00091644	0.01757276	-1.109359	3.03789431	1.75516007
**AFG3L2**	-0.1457699	0.03133141	-3.3760061	0.00091978	0.01760177	-1.1126844	3.03631524	1.75444355
**BABAM2**	0.09493573	0.02661819	3.37537362	0.00092176	0.01760468	-1.1146479	3.03538284	1.75437199
**TFB1M**	-0.2027956	-0.0079411	-3.3740202	0.000926	0.01765077	-1.1188484	3.03338812	1.75323641

**XPA**	0.24930798	-0.0142637	3.37279218	0.00094153	0.01791127	-1.0689782	3.02616794	1.74687367
**ETFRF1**	-0.2474949	0.07470351	-3.3676133	0.00094634	0.01796739	-1.1387129	3.02395361	1.74551509
**TAF3**	-0.2227422	-0.0437553	-3.36547	0.00095323	0.01806275	-1.1453507	3.02080052	1.74321607
**GFM1**	-0.1201458	0.03134596	-3.3559849	0.00098432	0.0186152	-1.1746827	3.00686428	1.73013222
**SRR**	0.39186997	0.00797215	3.35409451	0.0010028	0.01873067	-1.1263991	2.99878458	1.72744659
**GFM2**	-0.1647551	0.06616004	-3.3528207	0.0009949	0.01873067	-1.184452	3.00222158	1.72744659
**AKAP10**	0.13962806	0.02760182	3.35271399	0.00099526	0.01873067	-1.1847812	3.00206513	1.72744659
**ABHD12**	-0.1412588	0.04411531	-3.3517856	0.00099838	0.01873067	-1.187646	3.00070358	1.72744659
**AAAS**	-0.1165321	0.02617619	-3.3517453	0.00099852	0.01873067	-1.1877703	3.00064449	1.72744659
**EAPP**	0.16263259	0.04801957	3.35013606	0.00100396	0.01873067	-1.1927341	2.99828523	1.72744659
**FAM8A1**	-0.1908783	-0.0061261	-3.3501177	0.00100402	0.01873067	-1.1927908	2.99825828	1.72744659
**TMEM205**	-0.2164162	0.0365938	-3.3458286	0.00101865	0.01896698	-1.2060107	2.99197428	1.72200181
**ISCA1**	-0.190955	0.05703581	-3.3406349	0.00103664	0.01925004	-1.2219992	2.98437284	1.71556832
**COQ3**	-0.1482817	0.10455467	-3.3401402	0.00103837	0.01925004	-1.2235212	2.98364915	1.71556832
**PPT1**	-0.1535161	0.01265722	-3.3397187	0.00103984	0.01925004	-1.2248174	2.98303283	1.71556832
**LIMCH1**	0.47759449	-0.0843404	3.35322747	0.00104719	0.019312	-1.1374041	2.97997338	1.71417284
**DOCK4**	-0.2702809	-0.042318	-3.3413296	0.00104676	0.019312	-1.1654427	2.98015433	1.71417284
**AOC1**	0.71699624	-0.5619393	3.3584628	0.00105145	0.0193535	-0.9708446	2.97821134	1.7132404
**UACA**	-0.3032083	-0.0291071	-3.3328491	0.00106416	0.0195501	-1.2459271	2.97299405	1.70885112
**TIMM9**	-0.2083922	-0.0279861	-3.3322606	0.00106627	0.01955157	-1.2477338	2.97213475	1.70881827
**DOLPP1**	-0.3713214	0.02883551	-3.3378501	0.00107337	0.01962551	-1.1056116	2.96925126	1.70717903
**TAF12**	-0.1743102	0.00976261	-3.3331046	0.00107602	0.01962551	-1.1905322	2.9681778	1.70717903
**TK2**	-0.204515	0.05907103	-3.3294452	0.0010764	0.01962551	-1.2563728	2.96802559	1.70717903
**ANKRD50**	-0.1522631	-0.008797	-3.3274888	0.0010835	0.01971763	-1.2623723	2.96517165	1.70514529
**NDUFB3**	-0.1238507	0.0502374	-3.3254932	0.00109078	0.01981281	-1.2684891	2.9622617	1.70305399
**FAM83D**	0.58652006	-0.2137222	3.33346413	0.00110377	0.020011	-1.0380785	2.95712178	1.69873119
**SLC2A6**	-0.3517907	-0.0603031	-3.3192441	0.00111389	0.02015658	-1.2876222	2.95315809	1.69558307
**NEK3**	-0.2196575	-0.0287653	-3.3122422	0.00114032	0.02059624	-1.309023	2.94297294	1.68621197
**WDR48**	-0.1082764	0.05721356	-3.3085695	0.00115442	0.02081189	-1.3202327	2.93763688	1.68168843
**CTTN**	0.46106214	0.0125693	3.30426734	0.00117114	0.02107393	-1.3333496	2.931392	1.67625456
**PIP4K2C**	0.19950056	0.11228436	3.30304419	0.00117593	0.0211208	-1.3370762	2.92961761	1.67528966
**NDUFB7**	-0.1208459	0.01813771	-3.3000304	0.00118782	0.02129474	-1.3462533	2.92524764	1.67172769
**ATP10D**	0.21493848	0.00165008	3.30467402	0.00119863	0.02144858	-1.2053101	2.92131502	1.66860155
**NSUN4**	-0.1900342	0.04888437	-3.2949434	0.00120815	0.02157895	-1.3617268	2.91787822	1.66596975
**RDH14**	-0.1368356	0.09027222	-3.2899275	0.00122851	0.02190205	-1.3769633	2.91062029	1.65951533
**DL1**	0.31830615	-0.0807237	3.29244096	0.00123229	0.02192886	-1.3137885	2.90928694	1.658984
**SESN2**	-0.190686	-0.0371499	-3.2881493	0.00123581	0.02195095	-1.3823602	2.9080491	1.6585467
**SEC61A1**	-0.1371246	0.03798982	-3.2854277	0.00124705	0.02210993	-1.3906154	2.9041159	1.65541256
**MRPL57**	-0.1436542	-0.0082304	-3.2818765	0.00126186	0.02233152	-1.4013777	2.89898749	1.65108176
**TEFM**	-0.1688454	0.05516979	-3.2807898	0.00126643	0.02237127	-1.4046689	2.89741904	1.65030945
**ELP3**	0.08894516	0.00918391	3.2795449	0.00127168	0.02242292	-1.4084382	2.89562264	1.64930774
**ZNF395**	-0.4806005	0.02424698	-3.2842256	0.00130096	0.02279437	-1.171516	2.88573675	1.6421725
**SLC39A1**	-0.2922341	0.14436838	-3.2808333	0.00129693	0.02279437	-1.2764287	2.88708345	1.6421725
**SFXN5**	-0.1903052	0.03526079	-3.2727585	0.00130065	0.02279437	-1.4289643	2.88583876	1.6421725
**MMAA**	-0.1509966	0.09231454	-3.2724005	0.0013022	0.02279437	-1.4300459	2.88532313	1.6421725
**OXA1L**	-0.1443506	0.01135201	-3.2716135	0.0013056	0.02281254	-1.4324236	2.8841896	1.64182644
**HHAT**	-0.2571263	0.09969531	-3.2748581	0.00132272	0.02306985	-1.2941839	2.87853241	1.63695531
**CCDC127**	-0.154921	0.05626074	-3.2664758	0.00132802	0.02312053	-1.4479335	2.87679467	1.6360022
**CXCR2**	0.62515667	-0.0821615	3.28156035	0.00133125	0.02313501	-1.2842488	2.87573965	1.6357304
**RBM18**	0.18551386	0.05589247	3.26697859	0.00135548	0.02351372	-1.3266618	2.86790604	1.6286786
**NDUFAF7**	-0.1606271	-0.0163752	-3.2587763	0.0013623	0.02357415	-1.4711371	2.86572859	1.62756398
**SNRNP35**	0.21108806	0.12482205	3.25843051	0.00136385	0.02357415	-1.4721782	2.86523201	1.62756398
**GAMT**	0.17463963	0.05038132	3.25654159	0.0013724	0.02367937	-1.4778629	2.86252034	1.62562992
**DFFB**	-0.2095517	0.00439644	-3.2539856	0.00138404	0.02383753	-1.4855507	2.85885289	1.62273868
**SNRPB2**	0.06664891	0.02531694	3.25327821	0.00138727	0.0238507	-1.4876772	2.85783833	1.62249896
**OSGEPL1**	-0.2048458	0.01290678	-3.2515581	0.00141053	0.02420745	-1.4363892	2.85061687	1.61605095
**NUP155**	-0.0822974	0.05404023	-3.2467064	0.00141768	0.02428698	-1.5074153	2.84842029	1.61462645
**NDC1**	-0.1289331	0.00320219	-3.2456268	0.00142274	0.02430023	-1.5106545	2.84687442	1.61438956
**HDAC10**	0.16605584	0.03115721	3.24546538	0.0014235	0.02430023	-1.5111387	2.84664335	1.61438956
**GOT2**	-0.175271	0.03949643	-3.2425524	0.00143723	0.02449127	-1.5198737	2.84247441	1.61098861
**DUS2**	0.12714431	0.02412109	3.24187761	0.00144043	0.02450248	-1.521896	2.84150915	1.61078998
**TPD52**	0.51204024	-0.1384827	3.2489293	0.00145786	0.02466858	-1.2862093	2.83628314	1.60785576
**XPR1**	-0.1586834	0.00221795	-3.2416735	0.00145709	0.02466858	-1.4658318	2.836513	1.60785576
**MRPS30**	-0.1458834	-0.0026397	-3.2389665	0.0014543	0.02466858	-1.530617	2.83734633	1.60785576
**NEURL4**	-0.1301826	-0.0125895	-3.2350395	0.00147321	0.02488461	-1.5423702	2.83173532	1.6040692

**TCEANC**	0.33337234	0.11509514	3.26395172	0.00148843	0.02507313	-1.2772206	2.82727174	1.6007915
**SPATA2**	0.1503389	0.05893309	3.23863604	0.00148957	0.02507313	-1.4012227	2.82693911	1.6007915
**CNIH4**	0.1618233	0.05247962	3.23069784	0.00149438	0.02511034	-1.5553498	2.82553778	1.60014744
**BUD31**	-0.1407571	0.06742972	-3.2299089	0.00149826	0.02513171	-1.5577067	2.82441227	1.59977789
**ZNF561**	-0.3525411	0.01774705	-3.2404994	0.00152426	0.0255125	-1.1936317	2.81694084	1.59324696
**ST3GAL4**	-0.3597034	-0.0775139	-3.2242699	0.00152625	0.0255125	-1.5745387	2.81637338	1.59324696
**MRPL20**	-0.1261628	0.01435388	-3.2217745	0.00153879	0.02565675	-1.5819788	2.81281939	1.59079843
**TM9SF3**	-0.1139248	0.0321622	-3.2214954	0.0015402	0.02565675	-1.5828105	2.8124221	1.59079843
**CASK**	-0.2700962	-0.0594265	-3.2147879	0.00159107	0.02645834	-1.5455203	2.79831161	1.57743737
**EIF3F**	0.071688	0.05321337	3.20882533	0.00160543	0.02665118	-1.6205067	2.79440972	1.57428362
**CHCHD10**	-0.354825	-0.1121682	-3.2510514	0.00162575	0.02666728	-1.4624607	2.78894614	1.57402123
**BBS7**	0.31973798	0.11321771	3.21331659	0.00161539	0.02666728	-1.484767	2.79172128	1.57402123
**TIMM44**	-0.1231037	0.03436904	-3.2068026	0.00161607	0.02666728	-1.6265126	2.79153901	1.57402123
**NDUFB10**	-0.0999482	0.04189106	-3.2063373	0.00161853	0.02666728	-1.6278937	2.79087883	1.57402123
**TMCC1**	-0.1180065	0.03720287	-3.2058478	0.00162112	0.02666728	-1.6293464	2.79018444	1.57402123
**ARHGDIB**	0.16190864	-0.0317327	3.20517175	0.00162471	0.02666728	-1.6313526	2.78922545	1.57402123
**FITM2**	-0.2205653	0.07118222	-3.2050782	0.0016252	0.02666728	-1.63163	2.78909281	1.57402123
**SCARB2**	-0.1616139	0.07300917	-3.2022349	0.00164036	0.0268549	-1.6400629	2.78506139	1.57097642
**GLCE**	-0.1824434	0.01942941	-3.2012715	0.00164552	0.0268549	-1.6429187	2.78369607	1.57097642
**DTWD1**	-0.1821307	0.02902704	-3.20116	0.00164612	0.0268549	-1.6432494	2.78353797	1.57097642
**COQ6**	-0.1209862	0.06461292	-3.2007499	0.00164833	0.0268549	-1.6444646	2.78295695	1.57097642
**WWC3**	-0.2207956	-0.0567266	-3.1994676	0.00165524	0.02690376	-1.6482641	2.7811403	1.57018704
**MT-ND4**	-0.1805741	0.00031067	-3.1991587	0.0016569	0.02690376	-1.649179	2.78070283	1.57018704
**UBE2D2**	-0.1118802	0.01213615	-3.1962821	0.00167252	0.02711161	-1.6576967	2.77662988	1.56684462
**COX5B**	-0.1241248	0.04984912	-3.1947465	0.00168091	0.0271621	-1.662241	2.77445667	1.56603663
**FOXRED1**	-0.1536175	0.01958146	-3.1946814	0.00168126	0.0271621	-1.6624337	2.77436453	1.56603663
**SEC14L2**	0.57741108	0.161846	3.24147369	0.00169408	0.02732341	-1.3686147	2.77106611	1.56346506
**PNMA2**	-0.3417683	0.01564934	-3.2165047	0.00170145	0.02739644	-1.3912981	2.76918132	1.56230591
**DCAF17**	0.33243403	0.09231742	3.20481773	0.00173596	0.02780747	-1.3011332	2.76045945	1.55583852
**ZNF260**	-0.3994329	-0.051232	-3.211185	0.00173451	0.02780747	-1.3057028	2.76082331	1.55583852
**MTFMT**	-0.1497805	0.09410199	-3.1875314	0.00173851	0.02780747	-1.6257176	2.75982344	1.55583852
**CCNB2**	0.27968717	-0.1410113	3.18590616	0.00172997	0.02780747	-1.6883641	2.76196129	1.55583852
**ALG8**	-0.1546126	0.0729053	-3.1822397	0.00175071	0.02794309	-1.6991798	2.75678652	1.55372564
**PFN2**	0.35514548	0.07556857	3.18187535	0.00175278	0.02794309	-1.700254	2.7562725	1.55372564
**ZKSCAN8**	-0.1378229	-0.0147546	-3.1808391	0.00175869	0.02798783	-1.7033086	2.75481089	1.55303072
**NF2**	-0.1189375	-0.0522745	-3.1803664	0.00176139	0.02798783	-1.7047016	2.75414427	1.55303072
**NMD3**	0.09765138	0.02238027	3.17922969	0.0017679	0.02804511	-1.7080509	2.75254153	1.55214288
**PIR**	-0.4511235	-0.1932361	-3.1781401	0.00177417	0.02807773	-1.7112602	2.75100568	1.55163794
**NNT**	-0.1278475	0.05437512	-3.1778596	0.00177578	0.02807773	-1.7120862	2.75061034	1.55163794
**NSDHL**	-0.0994569	0.0549736	-3.176498	0.00178364	0.02815589	-1.7160951	2.74869171	1.55043069
**MEX3C**	-0.2380099	-0.0027123	-3.1906381	0.00178959	0.02816583	-1.4487951	2.7472456	1.55027741
**PDHA1**	-0.1356568	0.03987789	-3.1753817	0.00179011	0.02816583	-1.7193807	2.74711917	1.55027741
**CCDC32**	-0.1747659	-0.0244761	-3.1741111	0.00179751	0.02823607	-1.7231194	2.74532967	1.54919581
**CEACAM6**	0.73678658	-0.9493228	3.16876626	0.00182891	0.02868261	-1.738831	2.7378084	1.54238128
**AMD1**	0.3998674	-0.0623659	3.18590854	0.00183673	0.02871196	-1.4801504	2.73595426	1.5419372
**FAM210A**	-0.1314595	-0.0100895	-3.1675254	0.00183627	0.02871196	-1.7424753	2.73606359	1.5419372
**MBLAC1**	-0.379028	0.08394277	-3.1812089	0.00184122	0.02873555	-1.3645516	2.73489431	1.54158055
**HSPB1**	0.36363766	-0.1265074	3.16536187	0.00184917	0.02881303	-1.7488263	2.73302262	1.54041104
**UQCRQ**	-0.1180655	0.06819403	-3.1638087	0.00185849	0.02891146	-1.7533832	2.73084055	1.53893
**TMEM237**	0.2434093	-0.069106	3.18916431	0.00186421	0.02895386	-1.4704181	2.72950416	1.53829353
**ZNF785**	0.38405464	0.07500837	3.20017118	0.00187485	0.0290722	-1.470303	2.72703401	1.53652216
**GGCT**	0.18546566	0.05040207	3.15971312	0.00188326	0.02915573	-1.7653902	2.72509027	1.53527609
**NDUFS4**	-0.1042692	0.0311472	-3.1580525	0.00189339	0.02926559	-1.7702547	2.72276035	1.5336427
**GTF2B**	-0.1288842	-0.0460592	-3.1569414	0.00190019	0.0293238	-1.7735083	2.7212019	1.53277968
**ATP5MPL**	-0.2129361	0.10005456	-3.1589978	0.00190418	0.02933833	-1.7657325	2.72029246	1.53256456
**AASS**	-0.5569531	0.0732484	-3.1604396	0.0019187	0.02951489	-1.6287967	2.71699355	1.52995886
**ADRM1**	0.13191867	0.10710817	3.15185684	0.00193163	0.02966655	-1.788384	2.71407552	1.52773293
**LILRA3**	0.73007991	-0.4068944	3.15828313	0.00195624	0.02971329	-1.5334998	2.70857783	1.52704932
**C2orf42**	-0.153578	0.00843003	-3.1515104	0.00195285	0.02971329	-1.7307877	2.70933108	1.52704932
**HUS1**	0.10854482	0.08511562	3.15034581	0.00194107	0.02971329	-1.7928006	2.71195935	1.52704932
**NUBPL**	-0.2130338	0.05320994	-3.1499569	0.0019435	0.02971329	-1.7939371	2.71141482	1.52704932
**PANK1**	0.2904993	-0.0440398	3.14939646	0.00194702	0.02971329	-1.7955746	2.71063019	1.52704932
**ICMT**	-0.1937254	-0.0249236	-3.1493838	0.0019471	0.02971329	-1.7956116	2.71061244	1.52704932
**ABAT**	-0.2742132	0.02286733	-3.1481378	0.00195493	0.02971329	-1.7992511	2.70886847	1.52704932
**CPA3**	-0.5571303	-0.3089089	-3.1422247	0.00199251	0.03021665	-1.8165063	2.70059884	1.51975373

**SPRTN**	-0.2725388	0.05125637	-3.1701662	0.00200299	0.03032787	-1.6780247	2.69832085	1.51815805
**EPM2A**	0.22548584	-0.0419615	3.14592025	0.00200747	0.03034811	-1.7473226	2.69734992	1.51786837
**RPL22L1**	0.3327719	-0.2341072	3.1411534	0.00201887	0.03047261	-1.7608048	2.69489172	1.51609034
**SERPINB2**	0.54304972	-0.5475126	3.13715021	0.0020253	0.03052185	-1.8312914	2.69351133	1.51538908
**SASS6**	0.21772818	-0.0973789	3.13441274	0.00204319	0.03074346	-1.8392586	2.68969146	1.51224726
**DYNC2H1**	0.35854527	0.13552707	3.13821256	0.00206015	0.03095036	-1.7695826	2.68610147	1.50933427
**CTH**	0.34869061	0.12495724	3.13078056	0.00208706	0.03125726	-1.790781	2.68046552	1.50504915
**TSEN15**	-0.1449172	0.02629196	-3.1280186	0.00208555	0.03125726	-1.8578442	2.68077874	1.50504915
**CTCF**	-0.1311189	0.05851693	-3.1261542	0.00209806	0.03137329	-1.8632572	2.67818248	1.50343996
**PER1**	0.26272537	-0.048331	3.13020687	0.00211342	0.03145671	-1.715491	2.67501395	1.50228673
**AURKB**	0.33703961	-0.1482415	3.12705604	0.00211206	0.03145671	-1.8015232	2.67529418	1.50228673
**R3HDM2**	0.15726491	0.02690735	3.12696796	0.00211265	0.03145671	-1.8017771	2.67517194	1.50228673
**PTP4A1**	-0.3359688	0.02889313	-3.1322482	0.0021193	0.03149563	-1.7217122	2.67380719	1.50174965
**SETD6**	0.15122538	0.09668855	3.12082788	0.00213417	0.03166773	-1.8787051	2.67077192	1.49938305
**DRAP1**	-0.1070301	0.01998201	-3.1200807	0.00213928	0.0316948	-1.8808702	2.66973317	1.49901193
**PDE3A**	0.78980458	-0.2526162	3.12938362	0.00214469	0.03172633	-1.6148555	2.66863473	1.4985801
**NDUFS8**	-0.1113981	0.03511373	-3.118718	0.00214863	0.03173584	-1.884818	2.66783904	1.49844999
**TTYH2**	-0.2426792	0.01703516	-3.1168917	0.00220214	0.03242691	-1.7631661	2.65715536	1.48909443
**PEX16**	-0.1167082	0.03069751	-3.1113862	0.00219959	0.03242691	-1.9060317	2.65765869	1.48909443
**NUP93**	-0.0677512	0.04524965	-3.110105	0.00220861	0.03247258	-1.9097342	2.65588154	1.48848315
**PIGV**	-0.3187972	0.11329585	-3.1184591	0.00222022	0.03249466	-1.645442	2.65360488	1.48818806
**MRPL19**	-0.1402241	0.01050447	-3.1088839	0.00221723	0.03249466	-1.9132617	2.65418833	1.48818806
**BPHL**	-0.201358	-0.014181	-3.1086244	0.00221907	0.03249466	-1.9140113	2.65382847	1.48818806
**IDH3B**	-0.1015538	0.04110818	-3.1074618	0.00222732	0.03254928	-1.9173685	2.65221688	1.48745858
**C16orf54**	0.36201079	-0.0149146	3.11611057	0.00223677	0.03263788	-1.6520054	2.65037878	1.48627801
**TCEA2**	-0.2134056	-0.0485859	-3.1050155	0.00224477	0.03270521	-1.9244289	2.64882731	1.48538306
**ATP5PO**	-0.1125445	0.08517016	-3.1031972	0.00225783	0.03284577	-1.9296734	2.64630928	1.48352057
**ST8SIA4**	-0.2348522	-0.0362903	-3.1065426	0.00227845	0.03309579	-1.7828444	2.64236143	1.48022728
**CNNM4**	-0.1190443	0.0235332	-3.0964282	0.00230704	0.03341662	-1.9491739	2.63694469	1.47603748
**BLOC1S3**	0.10186193	0.07014072	3.09637094	0.00230746	0.03341662	-1.9493388	2.63686548	1.47603748
**EIF3E**	0.07549998	0.02734067	3.09468393	0.00231988	0.03354613	-1.9541929	2.63453398	1.47435758
**COX7C**	-0.3731071	-0.1477937	-3.0940728	0.0023244	0.0335611	-1.9559507	2.63368964	1.47416387
**RBFA**	-0.1455065	0.03231157	-3.0878541	0.0023708	0.03416825	-1.9738206	2.62510463	1.46637724
**ARMCX3**	-0.1564275	0.09405492	-3.0874916	0.00237353	0.03416825	-1.9748611	2.62460467	1.46637724
**PREPL**	-0.1396774	0.00851777	-3.0863896	0.00238185	0.03423694	-1.9780243	2.62308477	1.46550506
**EMID1**	-0.4791943	-0.0403555	-3.0930127	0.00240251	0.03437604	-1.823415	2.61933404	1.46374412
**EFEMP1**	0.40325919	-0.0522073	3.08934273	0.00240579	0.03437604	-1.8315194	2.61874258	1.46374412
**PRF1**	-0.3540887	-0.0072674	-3.0840764	0.00239941	0.03437604	-1.9846603	2.61989586	1.46374412
**EEFSEC**	0.08661693	0.05037407	3.08328005	0.00240548	0.03437604	-1.986944	2.61879839	1.46374412
**SLC25A20**	-0.1523249	0.08537589	-3.0822063	0.00241369	0.03443789	-1.9900222	2.617319	1.46296347
**COL5A1**	-0.5001032	-0.0584516	-3.0992095	0.00241869	0.03445836	-1.7232662	2.616419	1.46270544
**SH2D4A**	0.43012854	-0.3420318	3.08217602	0.00243334	0.03454581	-1.9876677	2.61379804	1.46160465
**ACAA1**	-0.1771225	0.04817082	-3.0802577	0.00242865	0.03454581	-1.9956061	2.61463523	1.46160465
**HSPD1**	-0.1183457	0.03741201	-3.079359	0.00243558	0.03454581	-1.9981802	2.61339797	1.46160465
**ARHGEF17**	0.21122541	0.04452111	3.07655895	0.00245728	0.03480251	-2.0061963	2.60954461	1.45838949
**GTPBP10**	-0.2063956	-0.0143733	-3.0707924	0.00250255	0.03537864	-2.0226846	2.60161705	1.45125885
**RNF13**	-0.1679189	0.04977079	-3.0704454	0.0025053	0.03537864	-2.0236758	2.60114038	1.45125885
**PRICKLE3**	0.46472678	0.08531352	3.07605817	0.00256633	0.03618749	-1.6479222	2.59068763	1.4414415
**ACOT2**	-0.1822571	0.01091269	-3.0620378	0.00257275	0.03622503	-2.047664	2.58960294	1.44099127
**FAM84B**	0.2170269	0.04281405	3.06150583	0.00257707	0.03623301	-2.0491798	2.58887373	1.44089562
**GTPBP1**	0.10119427	0.02015538	3.0605872	0.00258455	0.03624066	-2.0517969	2.58761472	1.44080394
**CDPF1**	-0.139121	0.0212038	-3.0605164	0.00258513	0.03624066	-2.0519985	2.58751773	1.44080394
**SLC9A1**	0.24456143	0.08168651	3.0600389	0.00258903	0.03624262	-2.0533586	2.58686339	1.44078038
**ISG15**	0.30337838	-0.1296891	3.05526135	0.00262832	0.0367394	-2.0669557	2.58032103	1.43486789
**SELENOK**	0.14864063	0.02763449	3.05996371	0.00263557	0.03678743	-1.9925925	2.57912471	1.43430052
**MAFF**	-0.3274167	-0.0772626	-3.0566873	0.00266585	0.03699581	-1.9232839	2.57416494	1.43184751
**ECI2**	-0.1718926	0.04379203	-3.0512965	0.00266135	0.03699581	-2.0782254	2.5748974	1.43184751
**MFF**	-0.0986786	0.01184683	-3.0510721	0.00266324	0.03699581	-2.0788631	2.57459048	1.43184751
**TMEM126B**	-0.206965	-0.0310386	-3.0510443	0.00266347	0.03699581	-2.078942	2.57455251	1.43184751
**PLK1**	0.274827	-0.2195391	3.05014831	0.00267099	0.03701394	-2.0814867	2.57332768	1.43163469
**KIF3B**	0.15634412	0.08759287	3.04920667	0.00267892	0.03707052	-2.0841605	2.57204072	1.43097127
**PSMB4**	0.06394647	0.03369125	3.04800555	0.00268906	0.037116	-2.0875699	2.57039954	1.43043885
**SRGAP3**	-0.4062559	-0.0602409	-3.0479063	0.0026899	0.037116	-2.0878517	2.5702639	1.43043885
**PUM2**	-0.0858742	-0.0068416	-3.0456534	0.00270903	0.03730381	-2.0942433	2.56718695	1.4282468
**GPR107**	-0.1866737	0.05691749	-3.0453927	0.00271125	0.03730381	-2.0949826	2.56683102	1.4282468

**CD93**	0.56266818	-0.3313938	3.05042643	0.00271858	0.03731176	-1.9407799	2.56565801	1.42815427
**MIS18A**	0.26307726	-0.1154695	3.04441853	0.00271956	0.03731176	-2.0977448	2.56550118	1.42815427
**PI4KA**	-0.0881075	0.04877215	-3.0411244	0.00274785	0.03764636	-2.1070795	2.56100657	1.424277
**ENY2**	-0.0840108	-0.0075307	-3.0401921	0.00275591	0.03770318	-2.1097197	2.55973518	1.42362207
**HOMER1**	0.63696245	0.06199463	3.05606732	0.00277021	0.03784516	-1.8423571	2.55748714	1.42198961
**SERPINB4**	-0.6004312	0.25334741	-3.0718327	0.00279217	0.03806157	-1.9479744	2.55405802	1.41951335
**DNPH1**	0.13805497	0.06286897	3.03582409	0.00279394	0.03806157	-2.1220802	2.55378232	1.41951335
**PHC1**	-0.2389592	-0.0267226	-3.0339276	0.00281061	0.03823457	-2.1274418	2.55119974	1.41754375
**CYP4V2**	-0.5672417	0.15740577	-3.0551372	0.00281869	0.03827521	-1.7204206	2.54995335	1.41708246
**MYO19**	-0.2447706	0.0166885	-3.0354317	0.00282153	0.03827521	-2.0622382	2.54951509	1.41708246
**HLCS**	-0.1200494	-0.0414822	-3.0316631	0.00283063	0.03834465	-2.1338402	2.54811749	1.41629525
**DMAC1**	-0.3412671	-0.1277219	-3.0306212	0.00283988	0.03841605	-2.1367824	2.54670003	1.41548733
**TOPORS**	-0.2118236	-0.0042627	-3.0273489	0.00286913	0.03871279	-2.146018	2.54225022	1.41214556
**MTCH2**	-0.1554953	0.03691912	-3.0272691	0.00286984	0.03871279	-2.1462431	2.54214175	1.41214556
**PSMG3**	0.17395382	0.03142604	3.02515775	0.00288887	0.03891495	-2.152197	2.53927271	1.4098835
**PTGR2**	0.29992769	0.02398993	3.02317182	0.00290686	0.03910278	-2.1577939	2.53657542	1.40779234
**MARCKSL1**	-0.4005925	-0.1779189	-3.0219651	0.00291785	0.0391959	-2.1611932	2.53493711	1.40675932
**YIPF4**	-0.1846284	-0.0185645	-3.024077	0.00292334	0.03921505	-2.1519139	2.53412057	1.40654723
**MRPL11**	-0.1247701	0.01988029	-3.0194008	0.00294132	0.03940148	-2.1684128	2.5314572	1.40448746
**AJUBA**	0.523038	0.23862692	3.04937263	0.00297306	0.03977135	-1.8795996	2.52679664	1.40042967
**RBM48**	0.16405087	-0.0038069	3.02055754	0.00298367	0.03985798	-2.0238165	2.5252498	1.39948477
**DCP2**	0.09947552	0.04304008	3.0142106	0.00298937	0.03987891	-2.1830085	2.52442071	1.39925677
**CSF1**	0.72048672	-0.0219252	3.04031466	0.00300498	0.03999087	-1.7571851	2.52215862	1.39803916
**UBQLN1**	0.10512073	-0.0050989	3.01242603	0.00300605	0.03999087	-2.1880218	2.52200338	1.39803916
**DPM3**	-0.1063029	0.02614219	-3.0105209	0.00302396	0.04017368	-2.1933708	2.51942398	1.39605837
**NDUFV3**	-0.1259666	0.0356465	-3.0097066	0.00303164	0.04022037	-2.1956565	2.51832172	1.3955539
**FLYWCH1**	-0.3825827	-0.0238842	-3.0217007	0.00303733	0.04024043	-1.7912319	2.51750822	1.39533737
**MCEMP1**	0.42728422	-0.2410708	3.00955978	0.00305844	0.04040905	-2.1346165	2.51450069	1.39352132
**MRPL54**	-0.1126208	-0.0026623	-3.0070866	0.00305649	0.04040905	-2.203006	2.51477707	1.39352132
**MALSU1**	-0.1689779	0.02534958	-3.0032175	0.00309352	0.04081672	-2.213849	2.50954674	1.38916188
**RSPRY1**	-0.0906499	0.0271179	-3.0022111	0.00310322	0.04088877	-2.2166673	2.50818715	1.388396
**NDUFAF2**	-0.1517002	-0.0147362	-3.00124	0.00311261	0.0409565	-2.2193861	2.50687545	1.38767719
**CSPP1**	0.21056041	-0.0428173	3.00778574	0.00313304	0.04116923	-1.9639615	2.50403344	1.38542727
**FNDC3A**	-0.144029	0.04541465	-2.9968202	0.00315566	0.0413593	-2.2317499	2.50090972	1.38342677
**ATP1B1**	-0.3240012	0.04527965	-2.9967768	0.00315609	0.0413593	-2.2318712	2.50085119	1.38342677
**DBT**	-0.1430191	0.09273937	-2.9960507	0.00316321	0.04139644	-2.2339008	2.49987177	1.38303703
**IKZF5**	0.36176503	0.11443108	3.01608315	0.00317044	0.04143484	-1.8208265	2.49888019	1.38263432
**PPP1R37**	0.11972005	0.0501819	2.99427441	0.00318071	0.04151274	-2.2388641	2.49747643	1.38181864
**ME2**	-0.1293975	0.03991237	-2.9930365	0.00319295	0.04159681	-2.2423213	2.49580783	1.38093998
**MRPL40**	-0.121288	0.01511411	-2.9927518	0.00319577	0.04159681	-2.2431165	2.49542401	1.38093998
**PIGA**	-0.1550402	0.05574918	-2.9917351	0.00320587	0.041672	-2.2459549	2.494054	1.38015567
**EIF2AK3**	-0.2022931	-0.0400687	-2.9903875	0.0032193	0.04179023	-2.2497157	2.49223865	1.37892524
**ZNF668**	0.10943475	0.0198328	2.98967005	0.00322647	0.04182701	-2.2517172	2.49127247	1.37854318
**FASTKD2**	-0.131564	0.02851741	-2.988644	0.00323675	0.04190395	-2.2545788	2.489891	1.37774504
**MANEA**	-0.1645909	0.03083073	-2.9881207	0.003242	0.04191573	-2.2560383	2.48918641	1.37762301
**PRMT7**	0.09778022	0.04014842	2.98760618	0.00324718	0.04192641	-2.2574727	2.48849391	1.37751228
**TXNDC9**	-0.1283513	-0.0516046	-2.9870879	0.0032524	0.04193767	-2.2589176	2.48779633	1.37739569
**SLC27A4**	-0.1208299	0.00420073	-2.98582	0.0032652	0.04204652	-2.262451	2.48609033	1.37626992
**NDUFA11**	-0.1365708	0.04353495	-2.9852943	0.00327052	0.04205889	-2.2639157	2.48538312	1.37614215
**MUT**	-0.1269577	0.09084399	-2.9830437	0.00329339	0.04229662	-2.2701835	2.48235662	1.37369432
**MLYCD**	-0.1597055	0.03663204	-2.9793283	0.00333147	0.04272878	-2.2805217	2.47736387	1.36927948
**ALDH18A1**	-0.1689884	0.01374015	-2.9778641	0.00334659	0.04286568	-2.2845927	2.47539752	1.36789027
**PBK**	0.37618543	-0.0957354	2.97734504	0.00335196	0.04287757	-2.2860354	2.47470067	1.36776979
**ZNF219**	-0.3200982	-0.0605345	-2.979249	0.00335922	0.0429135	-2.218714	2.47376132	1.36740604
**ADARB1**	-0.1602048	0.03650179	-2.9744342	0.00338225	0.04313063	-2.2941218	2.47079436	1.36521421
**DCP1A**	0.08939319	0.05366002	2.97415559	0.00338516	0.04313063	-2.2948956	2.47042056	1.36521421
**DSC3**	0.55496853	-0.1783047	3.004503	0.00342045	0.0435072	-1.9881636	2.46591652	1.36143891
**STK35**	-0.1504198	0.01545622	-2.9756312	0.00342751	0.0435072	-2.1473613	2.46502102	1.36143891
**MRPL9**	-0.1183392	0.02130233	-2.9697504	0.00343151	0.0435072	-2.3071191	2.46451459	1.36143891
**NBEAL1**	0.22509669	0.05211025	2.96922623	0.00343706	0.0435072	-2.3085725	2.46381228	1.36143891
**RPP30**	0.1416692	0.07488779	2.96920674	0.00343727	0.0435072	-2.3086265	2.46378617	1.36143891
**GADD45GIP1**	-0.1076221	0.00304091	-2.9687132	0.00344251	0.04351638	-2.3099948	2.46312496	1.36134726
**PGS1**	-0.147153	0.0917242	-2.968176	0.00344822	0.04353148	-2.3114837	2.46240546	1.36119659
**NRF1**	-0.0924013	0.0652034	-2.9670843	0.00345985	0.04362118	-2.3145091	2.46094335	1.36030255
**AFDN**	0.29558421	-0.1756091	2.96916972	0.00346512	0.04362372	-2.2465118	2.46028175	1.36027727

**CEBPG**	-0.2931525	-0.2137946	-2.9662185	0.00346909	0.04362372	-2.3169077	2.45978414	1.36027727
**CDCA2**	0.33345168	-0.1035019	2.96507957	0.00348129	0.04372013	-2.3200619	2.45825961	1.3593186
**GNB1L**	-0.1450378	-0.0096284	-2.9657573	0.00350166	0.04391876	-2.2559039	2.45572583	1.35734994
**PLEKHF2**	0.13729779	0.04390789	2.96247851	0.0035093	0.0439574	-2.3272616	2.45477955	1.35696805
**FAAP100**	0.12083275	-0.0435149	2.96197761	0.00351472	0.04396815	-2.3286474	2.45410965	1.35686179
**TEDC1**	0.33869002	-0.0045862	2.97656243	0.00353333	0.044041	-1.9009597	2.45181562	1.35614279
**COX4I1**	-0.1002627	0.03969181	-2.9605645	0.00353004	0.044041	-2.3325557	2.45222026	1.35614279
**NDUFA9**	-0.1134975	0.02921119	-2.9601784	0.00353424	0.044041	-2.3336235	2.45170406	1.35614279
**WDCP**	0.09203666	0.02724994	2.95684218	0.0035707	0.04443288	-2.3428433	2.44724632	1.35229555
**HSD17B8**	-0.2393137	0.07287203	-2.9564601	0.0035749	0.04443288	-2.3438986	2.44673606	1.35229555
**ZNF710**	0.26246491	0.08982748	2.9605196	0.00358995	0.04456255	-2.1885508	2.44491116	1.35102995
**ABCC3**	-0.261466	-0.0333935	-2.9529203	0.00361401	0.04480349	-2.3536698	2.4420108	1.34868816
**EIF1AX**	-0.2066524	0.00744308	-2.9517683	0.00362682	0.0449046	-2.3568474	2.44047398	1.34770921
**GALNT10**	-0.1862786	0.01493963	-2.9511656	0.00363354	0.04493012	-2.3585095	2.43967005	1.34746243
**TM9SF4**	-0.1280707	0.00402548	-2.9505907	0.00363996	0.04495187	-2.3600944	2.43890347	1.34725227
**GNPTG**	-0.1394343	-0.0064602	-2.947011	0.00368017	0.04539035	-2.3699583	2.43413199	1.34303651
**CCNQ**	-0.1634846	0.04806634	-2.9471358	0.00370738	0.04566758	-2.3069874	2.43093243	1.34039196
**TBC1D16**	-0.361122	-0.0406741	-2.9684916	0.00373172	0.04590068	-2.1726722	2.42809103	1.33818092
**RPL27A**	0.0939995	0.05758223	2.94187914	0.00373853	0.04590068	-2.3840798	2.42729936	1.33818092
**CSF1R**	-0.3446546	-0.189665	-2.9416997	0.00374058	0.04590068	-2.3845732	2.4270606	1.33818092
**ABCF2**	0.10613658	-0.0595851	2.94039996	0.00375551	0.04602523	-2.3881461	2.42533158	1.33700408
**ITPR2**	-0.219271	-0.0054058	-2.9385493	0.00377685	0.04611776	-2.393231	2.42287066	1.3361318
**ENSA**	0.14630096	0.0467091	2.93807315	0.00378236	0.04611776	-2.3945388	2.42223768	1.3361318
**UBAC2**	-0.1415367	-0.0348949	-2.937906	0.00378429	0.04611776	-2.394998	2.42201545	1.3361318
**NDUFS6**	-0.1100036	0.03547755	-2.9377265	0.00378637	0.04611776	-2.3954908	2.4217769	1.3361318
**MRPL10**	-0.1385598	0.04261247	-2.9376753	0.00378696	0.04611776	-2.3956314	2.42170885	1.3361318
**TSR3**	-0.1429344	-0.0574864	-2.9364186	0.00380155	0.04623707	-2.3990818	2.42003877	1.33500972
**RIOK3**	0.18215326	0.0965991	2.93410556	0.00382854	0.04650669	-2.405429	2.41696629	1.33248456
**JUN**	0.52271562	-0.1076878	2.9371158	0.00392591	0.04760487	-2.2534231	2.40605946	1.32234865
**TTC13**	-0.1215384	0.02885747	-2.9256409	0.00392882	0.04760487	-2.4286183	2.40573782	1.32234865
**GSS**	0.07798418	0.02554694	2.92510511	0.00393525	0.04762292	-2.4300842	2.40502787	1.32218396
**PDE5A**	1.01546849	-0.5384247	2.93571812	0.00395264	0.0477136	-1.9979546	2.40311327	1.32135782
**SLX4**	0.14460174	0.00532604	2.92624212	0.00395138	0.0477136	-2.3639627	2.40325144	1.32135782
**CPT2**	-0.1411665	0.0638981	-2.9230434	0.00396007	0.0477436	-2.4357222	2.40229706	1.32108481
**TRIM3**	-0.223114	0.02585171	-2.9261388	0.00398622	0.04793901	-2.2815722	2.39943876	1.3193109
**CYP20A1**	-0.0965629	0.0852124	-2.9212177	0.00398217	0.04793901	-2.440712	2.39987999	1.3193109
**AMACR**	-0.2473106	0.00054555	-2.9200291	0.00399662	0.04800425	-2.4439589	2.39830704	1.31872035
**BAHD1**	-0.1466632	0.02192735	-2.9157114	0.00404951	0.04856814	-2.455744	2.39259699	1.31364849
**GPN3**	0.09002955	0.03098682	2.91537688	0.00405364	0.04856814	-2.4566565	2.39215484	1.31364849
**NQO1**	0.29661921	0.02180819	2.91417215	0.00406853	0.04868602	-2.4599416	2.39056288	1.31259569
**GFPT1**	0.15781385	0.04775397	2.91244769	0.00408992	0.04884854	-2.4646419	2.38828499	1.31114842
**RETN**	0.52999003	-0.6169197	2.91226164	0.00409224	0.04884854	-2.4651489	2.38803929	1.31114842
**ARL4C**	-0.3045749	-0.0392502	-2.9375294	0.00410064	0.04888834	-2.2546763	2.38714845	1.31079474
**EIF3A**	0.08000229	-0.0096736	2.91020242	0.00411793	0.04895546	-2.4707579	2.38532066	1.3101989
**N6AMT1**	0.17518845	0.05457787	2.90996516	0.0041209	0.04895546	-2.471404	2.38500751	1.3101989
**TMEM230**	-0.1738028	-0.0325735	-2.9099179	0.0041215	0.04895546	-2.4715327	2.38494511	1.3101989
**MRPL12**	0.42564741	0.05550781	2.94418712	0.00416604	0.04930237	-2.0359001	2.38027686	1.30713224
**GCDH**	-0.1035655	0.06776124	-2.9067388	0.00416149	0.04930237	-2.4801844	2.38075113	1.30713224
**COPS4**	0.05520897	0.04774815	2.906402	0.00416575	0.04930237	-2.4811005	2.38030701	1.30713224
**TRMT10C**	-0.1263695	0.06778309	-2.9058482	0.00417276	0.04932138	-2.4826067	2.37957682	1.30696474
**TAF1C**	0.16256088	0.07347134	2.9023732	0.00421699	0.0497832	-2.4920513	2.3749974	1.30291722
**ABHD11**	-0.2017997	-0.0056826	-2.9011093	0.00423318	0.04988321	-2.4954839	2.37333286	1.30204557
**TRMT61B**	-0.158707	0.02567069	-2.9006534	0.00423904	0.04988321	-2.4967217	2.37273258	1.30204557
**RABEPK**	-0.1072109	0.05328078	-2.9005026	0.00424098	0.04988321	-2.4971313	2.37253395	1.30204557

Supplemental Table 6: Drugs with significantly different DSS between both cohorts.

Drug name	Drug class	P value	Frozen mean	Fresh mean	Difference	Standard Error	q value
Erastin	Metabolic modifier	<0.000001	12.85	4.909	7.943	1.068	<0.000001
Amcasertib	Kinase inhibitor	<0.000001	8.203	3.813	4.389	0.6103	<0.000001
Dinaciclib	Kinase inhibitor	<0.000001	27.79	24.39	3.402	0.6207	0.000025
GSK-J4	Differentiating/ epigenetic modifier	<0.000001	9.873	4.351	5.522	0.9997	0.000029
CCT196969	Kinase inhibitor	0.000003	8.919	4.842	4.077	0.8257	0.000283
S-63845	Apoptotic modulator	0.000004	15.79	7.884	7.910	1.622	0.000283
Plicamycin	Conventional chemotherapy	0.000004	16.34	9.249	7.093	1.486	0.000283
Fedratinib		0.000022	17.59	14.42	3.171	0.7147	0.001307
Auranofin	Conventional chemotherapy	0.000023	9.364	7.160	2.204	0.5062	0.001307
Gilteritinib	Kinase inhibitor	0.000028	6.160	3.790	2.370	0.5503	0.001418
Ralimetinib	Kinase inhibitor	0.000034	5.384	2.123	3.262	0.7548	0.001472
LY3009120	Kinase inhibitor	0.000035	16.38	12.24	4.136	0.9575	0.001472
PF-03758309	Kinase inhibitor	0.000039	22.79	18.07	4.720	1.102	0.001550
Valproic acid	Differentiating/ epigenetic modifier	0.000044	0.8776	0.1776	0.7000	0.1670	0.001605
Pacritinib	Kinase inhibitor	0.000055	17.42	13.74	3.687	0.8916	0.001887
BCI	Other	0.000123	21.59	17.85	3.739	0.9390	0.003790
Copanlisib	Kinase inhibitor	0.000126	13.00	8.744	4.253	1.070	0.003790
Navitoclax	Apoptotic modulator	0.000165	18.13	12.80	5.326	1.382	0.004601
VLX1570	Other	0.000176	30.18	26.29	3.890	1.001	0.004601
dBET1	Differentiating/ epigenetic modifier	0.000180	27.13	23.27	3.856	0.9946	0.004601
Ruboxistaurin	Kinase inhibitor	0.000220	9.852	8.037	1.815	0.4808	0.005348
NVP-SHP099	Other	0.000272	7.142	4.501	2.640	0.7016	0.006315

Supplemental Table 7: HiRIEF fractions gradient length.

**Sample**	**Gradient length**	**Sample**	**Gradient length**
fraction_01	50 min	fraction_37	50 min
fraction_02	50 min	fraction_38	70 min
fraction_03	70 min	fraction_39	70 min
fraction_04	70 min	fraction_40	50 min
fraction_05	90 min	fraction_41	50 min
fraction_06	90 min	fraction_42	50 min
fraction_07	110 min	fraction_43	accumulate in trap column
fraction_08	110 min	fraction_44	accumulate in trap column
fraction_09	110 min	fraction_45	accumulate in trap column
fraction_10	110 min	fraction_46	accumulate in trap column
fraction_11	110 min	fraction_47	accumulate in trap column
fraction_12	90 min	fraction_48	accumulate in trap column
fraction_13	90 min	fraction_49	accumulate in trap column
fraction_14	90 min	fraction_50	50 min
fraction_15	90 min	fraction_51	70 min
fraction_16	70 min	fraction_52	70 min
fraction_17	50 min	fraction_53	accumulate in trap column
fraction_18	50 min	fraction_54	accumulate in trap column
fraction_19	50 min	fraction_55	accumulate in trap column
fraction_20	accumulate in trap column	fraction_56	accumulate in trap column
fraction_21	accumulate in trap column	fraction_57	accumulate in trap column
fraction_22	accumulate in trap column	fraction_58	accumulate in trap column
fraction_23	accumulate in trap column	fraction_59	accumulate in trap column
fraction_24	accumulate in trap column	fraction_60	accumulate in trap column
fraction_25	accumulate in trap column	fraction_61	accumulate in trap column
fraction_26	accumulate in trap column	fraction_62	accumulate in trap column
fraction_27	50 min	fraction_63	accumulate in trap column
fraction_28	50 min	fraction_64	50 min
fraction_29	70 min	fraction_65	50 min
fraction_30	50 min	fraction_66	70 min
fraction_31	50 min	fraction_67	accumulate in trap column
fraction_32	accumulate in trap column	fraction_68	accumulate in trap column
fraction_33	accumulate in trap column	fraction_69	accumulate in trap column

fraction_34	accumulate in trap column	fraction_70	accumulate in trap column
fraction_35	accumulate in trap column	fraction_71	50 min
fraction_36	50 min	fraction_72	50 min

Supplemental Table 8: List of drugs used in custom drug plates for flow cytometry-based drug testing.

**Compound name**	**Concentration range (nM)**	**Manufacturer**	**Catalog number**
Amcasertib	1 - 10000	SelleckChem	S8572
Erastin	1 - 10000	Tocris	5449/10
Filanesib	0.1 - 1000	Medchem Express	HY-15187
NVP-LCL161	0.25 - 2500	SelleckChem	S7009
Paclitaxel	0.1 - 1000	Sigma	T7191
Plicamycin	1 - 10000	Medchem Express	HY-A0122
Pracinostat	1 - 10000	SelleckChem	S1515
S-63845	0.1 - 1000	Medchem Express	HY-100741
VLX1570	1 - 10000	SelleckChem	S8288

Supplemental Table 9: List of antibodies used for flow cytometry.

**Description**	**Conjugate**	**Clone**	**Host species**	**Dilution**	**Manufacturer**
CD3	BV510	HIT3a	Mouse IgG2a, k	1:40	BD Biosciences
CD11b	PE-Cy7	ICRF44	Mouse IgG1, k	1:40	BD Biosciences
CD14	FITC	MjP9	Mouse BALB/c IgG2b, k	1:10	BD Biosciences
CD19	Alexa Fluor 700	HIB19	Mouse IgG1, k	1:40	BD Biosciences
CD34	APC	8G12	Mouse BALB/c IgG1, k	1:40	BD Biosciences
CD38	BV421	HIT2	Mouse IgG1, k	1:40	BD Biosciences
CD45	BV786	HI30	Mouse IgG1, k	1:40	BD Biosciences
CD56	PE	B159	Mouse IgG1, k	1:10	BD Biosciences
CD64	APC-H7	10.1	Mouse BALB/c IgG1, k	1:40	BD Biosciences
CD66b	Alexa Fluor 700	G10F5	Mouse IgM, k	1:40	Biolegend
CD117	BV605	104D2	Mouse BALB/c IgG1	1:40	BD Biosciences
p53	PE	DO-7	Mouse IgG2b	1:10	BD Biosciences
Viability dye	7AAD			1:40	BD Biosciences

Supplemental references

1 Baccelli, I. *et al.* A novel approach for the identification of efficient combination therapies in primary human acute myeloid leukemia specimens. *Blood Cancer J* **7**, e529 (2017). https://doi.org/10.1038/bcj.2017.10

2 Bennett, T. A. *et al.* Pharmacological profiles of acute myeloid leukemia treatments in patient samples by automated flow cytometry: a bridge to individualized medicine. *Clin Lymphoma Myeloma Leuk* **14**, 305-318 (2014). https://doi.org/10.1016/j.clml.2013.11.006

3 Bhatt, S. *et al.* Reduced Mitochondrial Apoptotic Priming Drives Resistance to BH3 Mimetics in Acute Myeloid Leukemia. *Cancer Cell* **38**, 872-890 e876 (2020). https://doi.org/10.1016/j.ccell.2020.10.010

4 Collignon, A. *et al.* A chemogenomic approach to identify personalized therapy for patients with relapse or refractory acute myeloid leukemia: results of a prospective feasibility study. *Blood Cancer J* **10**, 64 (2020). https://doi.org/10.1038/s41408-020-0330-5

5 Dembitz, V. *et al.* 5-aminoimidazole-4-carboxamide ribonucleoside induces differentiation in a subset of primary acute myeloid leukemia blasts. *BMC Cancer* **20**, 1090 (2020). https://doi.org/10.1186/s12885-020-07533-6

6 Faraoni, I. *et al.* BRCA1, PARP1 and γH2AX in acute myeloid leukemia: Role as biomarkers of response to the PARP inhibitor olaparib. *Biochim Biophys Acta* **1852**, 462-472 (2015). https://doi.org/10.1016/j.bbadis.2014.12.001

7 Frismantas, V. *et al.* Ex vivo drug response profiling detects recurrent sensitivity patterns in drug-resistant acute lymphoblastic leukemia. *Blood* **129**, e26-e37 (2017). https://doi.org/10.1182/blood-2016-09-738070

8 Kamens, J. L. *et al.* Proteasome inhibition targets the KMT2A transcriptional complex in acute lymphoblastic leukemia. *Nat Commun* **14**, 809 (2023). https://doi.org/10.1038/s41467-023-36370-x

9 Knorr, K. L. *et al.* Assessment of Drug Sensitivity in Hematopoietic Stem and Progenitor Cells from Acute Myelogenous Leukemia and Myelodysplastic Syndrome

Ex Vivo. *Stem Cells Transl Med* **6**, 840-850 (2017). https://doi.org/10.5966/sctm.2016-0034

10 Kornauth, C. *et al.* Functional Precision Medicine Provides Clinical Benefit in Advanced Aggressive Hematologic Cancers and Identifies Exceptional Responders. *Cancer Discov* **12**, 372-387 (2022). https://doi.org/10.1158/2159-8290.cd-21-0538

11 Kurtz, S. E. *et al.* Molecularly targeted drug combinations demonstrate selective effectiveness for myeloid- and lymphoid-derived hematologic malignancies. *Proc Natl Acad Sci U S A* **114**, E7554-E7563 (2017). https://doi.org/10.1073/pnas.1703094114

12 Kuusanmäki, H. *et al.* Phenotype-based drug screening reveals association between venetoclax response and differentiation stage in acute myeloid leukemia. *Haematologica* **105**, 708-720 (2020). https://doi.org/10.3324/haematol.2018.214882

13 Lamble, A. J. *et al.* Reversible suppression of T cell function in the bone marrow microenvironment of acute myeloid leukemia. *Proc Natl Acad Sci U S A* **117**, 14331-14341 (2020). https://doi.org/10.1073/pnas.1916206117

14 Leung, G. M. K. *et al.* Distinct mutation spectrum, clinical outcome and therapeutic responses of typical complex/monosomy karyotype acute myeloid leukemia carrying TP53 mutations. *Am J Hematol* **94**, 650-657 (2019). https://doi.org/10.1002/ajh.25469

15 Lin, L. *et al.* Ex-vivo drug testing predicts chemosensitivity in acute myeloid leukemia. *J Leukoc Biol* **107**, 859-870 (2020). https://doi.org/10.1002/jlb.5a0220-676rr

16 Malani, D. *et al.* Implementing a Functional Precision Medicine Tumor Board for Acute Myeloid Leukemia. *Cancer Discov* **12**, 388-401 (2022). https://doi.org/10.1158/2159-8290.cd-21-0410

17 Metts, J. *et al.* Imipramine blue sensitively and selectively targets FLT3-ITD positive acute myeloid leukemia cells. *Sci Rep* **7**, 4447 (2017). https://doi.org/10.1038/s41598-017-04796-1

18 Onecha, E. *et al.* Improving the prediction of acute myeloid leukaemia outcomes by complementing mutational profiling with ex vivo chemosensitivity. *Br J Haematol* **189**, 672-683 (2020). https://doi.org/10.1111/bjh.16432

19 Pan, R. *et al.* Selective BCL-2 inhibition by ABT-199 causes on-target cell death in acute myeloid leukemia. *Cancer Discov* **4**, 362-375 (2014). https://doi.org/10.1158/2159-8290.cd-13-0609

20 Pei, S. *et al.* Monocytic Subclones Confer Resistance to Venetoclax-Based Therapy in Patients with Acute Myeloid Leukemia. *Cancer Discov* **10**, 536-551 (2020). https://doi.org/10.1158/2159-8290.cd-19-0710

21 Ramsey, H. E. *et al.* BET Inhibition Enhances the Antileukemic Activity of Low-dose Venetoclax in Acute Myeloid Leukemia. *Clin Cancer Res* **27**, 598-607 (2021). https://doi.org/10.1158/1078-0432.ccr-20-1346

22 Simon, L. *et al.* Chemogenomic Landscape of RUNX1-mutated AML Reveals Importance of RUNX1 Allele Dosage in Genetics and Glucocorticoid Sensitivity. *Clin Cancer Res* **23**, 6969-6981 (2017). https://doi.org/10.1158/1078-0432.ccr-17-1259

23 Spinner, M. A. *et al.* Ex vivo drug screening defines novel drug sensitivity patterns for informing personalized therapy in myeloid neoplasms. *Blood Adv* **4**, 2768-2778 (2020). https://doi.org/10.1182/bloodadvances.2020001934

24 Swords, R. T. *et al.* Ex-vivo sensitivity profiling to guide clinical decision making in acute myeloid leukemia: A pilot study. *Leuk Res* **64**, 34-41 (2018). https://doi.org/10.1016/j.leukres.2017.11.008

25 Tavor, S. *et al.* Dasatinib response in acute myeloid leukemia is correlated with FLT3/ITD, PTPN11 mutations and a unique gene expression signature. *Haematologica* **105**, 2795-2804 (2020). https://doi.org/10.3324/haematol.2019.240705

26 Tyner, J. W. *et al.* Functional genomic landscape of acute myeloid leukaemia. *Nature* **562**, 526-531 (2018). https://doi.org/10.1038/s41586-018-0623-z
